# Transcriptomic analysis reveals an association of *FCGBP* with Parkinson’s disease

**DOI:** 10.1038/s41531-022-00415-7

**Published:** 2022-11-12

**Authors:** Pilar Gómez-Garre, María Teresa Periñán, Silvia Jesús, Maria Giulia Bacalini, Paolo Garagnani, Brit Mollenhauer, Chiara Pirazzini, Federica Provini, Claudia Trenkwalder, Claudio Franceschi, Pablo Mir, on behalf of the PROPAG-AGEING consortium

**Affiliations:** 1 Unidad de Trastornos del Movimiento, Servicio de Neurología y Neurofisiología Clínica, Instituto de Biomedicina de Sevilla, Hospital Universitario Virgen del Rocío/CSIC/Universidad de Sevilla, Seville, Spain; 2 Centro de Investigación Biomédica en Red sobre enfermedades Neurodegenerativas (CIBERNED), Madrid, Spain; 3 IRCCS Instituto delle Scienze Neurologiche di Bologna, Bologna, Italy; 4 Department of Experimental, Diagnostic, and Specialty Medicine (DIMES), University of Bologna, Bologna, Italy; 5 Department of Neurology, University Medical Center Göttingen, Göttingen, Germany; 6 Paracelsus-Elena-Klinik, Kassel, Germany; 7 Department of Neurosurgery, University Medical Center Göttingen, Göttingen, Germany; 8 Department of Biomedical and NeuroMotor Sciences (DiBiNeM), University of Bologna, Bologna, Italy; 9 IRCCS Institute of Neurological Sciences of Bologna, Bologna, Italy; 10 Laboratory of Systems Medicine of Healthy Aging and Department of Applied Mathematics, Lobachevsky University, Nizhny Novgorod, Russia; 11 Department of Medicine, Faculty of Medicine, University of Seville, Seville, Spain

**Keywords:** Parkinson's disease, Next-generation sequencing

## Abstract

Transcriptomics in Parkinson’s disease (PD) offers new insights into the molecular mechanism of PD pathogenesis. Several pathways, such as inflammation and protein degradation, have been identified by differential gene expression analysis. Our aim was to identify gene expression differences underlying the disease etiology and the discovery of pre-symptomatic risk biomarkers for PD from a multicenter study in the context of the PROPAG-AGEING project. We performed RNA sequencing from 47 patients with de novo PD, 10 centenarians, and 65 healthy controls. Using identified differentially expressed genes, functional annotations were assigned using gene ontology to unveil significant enriched biological processes. The expression of 16 selected genes was validated using OpenArray® assays and samples from independent cohorts of 201 patients with advanced PD, 340 healthy siblings of PD patients, and 177 healthy controls. Differential gene expression analysis identified higher *FCGBP* expression in patients with de novo PD compared with healthy controls and compared with centenarians. Furthermore, *FCGBP* showed no differences in terms of population origin or aging process. The increased *FCGBP* expression was validated in patients with advanced PD and their siblings. Thus, we provided evidence for an upregulation of *FCGBP* mRNA levels not only in patients with PD but also in individuals at putative higher risk of PD, suggesting that it could be important in gut–brain PD interaction, mediating the connection between microbiota and intestinal inflammatory processes, as well as neuroinflammation and neurodegeneration.

## Introduction

Parkinson’s disease (PD) is a frequent neurodegenerative disorder caused by a progressive loss of neurons producing the neurotransmitter dopamine. The etiopathogenesis of PD is multifactorial and includes environmental and genetic factors, with age its main risk factor. Despite recent advances, there is a lack of knowledge regarding the heterogeneous processes that underlie the initiation and progression of PD, and biomarkers with possible clinical utility are few, partly due to the disorder’s intrinsic complexity and the multiple interacting factors.

Clinically, PD is defined as a movement disorder, although its nonmotor symptoms can be severe, with a long prodromal phase. Pathologically, there is selective loss of dopaminergic neurons in the substantia nigra, resulting in dopaminergic degeneration associated with the emergence of Lewy bodies, consisting mainly of aggregated α-synuclein (α-Syn)^1^. Common cellular and molecular converging pathways among the various PD phenotypes include mitochondrial dysfunction, impaired autophagy–lysosomal function, oxidative stress, and neuroinflammation^2^, which lead to the accumulation and spread of aggregated α-Syn, resulting in neurodegeneration. However, the neuropathology of PD also shows considerable heterogeneity.

The PD diagnosis is based on the presence of several clinical (mainly motor) features, and no current cure or therapeutic agent can successfully slow its progression. Investigating the pathophysiological and molecular mechanisms of PD could lead to the development of specific treatment guidelines and selective use of drugs. Growing evidence suggests that PD is a multisystem disorder rather than merely a dopaminergic motor syndrome with central and peripheral clinical manifestations^3^.

In the era of personalized medicine and molecular diagnosis, transcriptomics is emerging as an important tool in disease diagnosis and prognosis. In recent years, certain PD-related expression signatures have been reported. Although individual studies have highlighted several genes with altered expression in patients with PD, there is a lack of interstudy concordance, given the differing scopes, aims, and methodologies applied. A descriptive review of transcriptomic PD studies reported the concordance of several pathways, such as mitochondrial function, protein degradation, and inflammation, identified in blood and brain tissues, supporting the hypothesis that the disease process is systemic and not restricted to neurological tissues^4^. The comparison of different PD tissues yields few significant differentially expressed genes and pathways, suggesting that divergent gene expression profiles in distinct cell lineages create excessive transcriptomic noise for detecting significant signals^5^.

In this study, we aimed to provide a better definition of the expression differences between patients with PD and healthy controls using a dual approximation. We analyzed differentially expressed genes associated with PD and aging by RNA sequencing in untreated patients with PD in early stages of disease progression and in healthy controls, including centenarians as “supercontrols” indicative of successful aging. We validated our findings by analyzing candidate genes using OpenArray assays in patients with advanced PD and in healthy individuals at putative higher risk of developing PD, such as siblings of PD patients^6,7^. Our aim was to provide new insights into the disease etiology and to discover presymptomatic PD risk biomarkers that could improve the clinical management and enhance treatment.

## Results

### Discovery stage

#### Differentially expressed genes in discovery cohorts via RNA-Seq

**Fig. 1:**
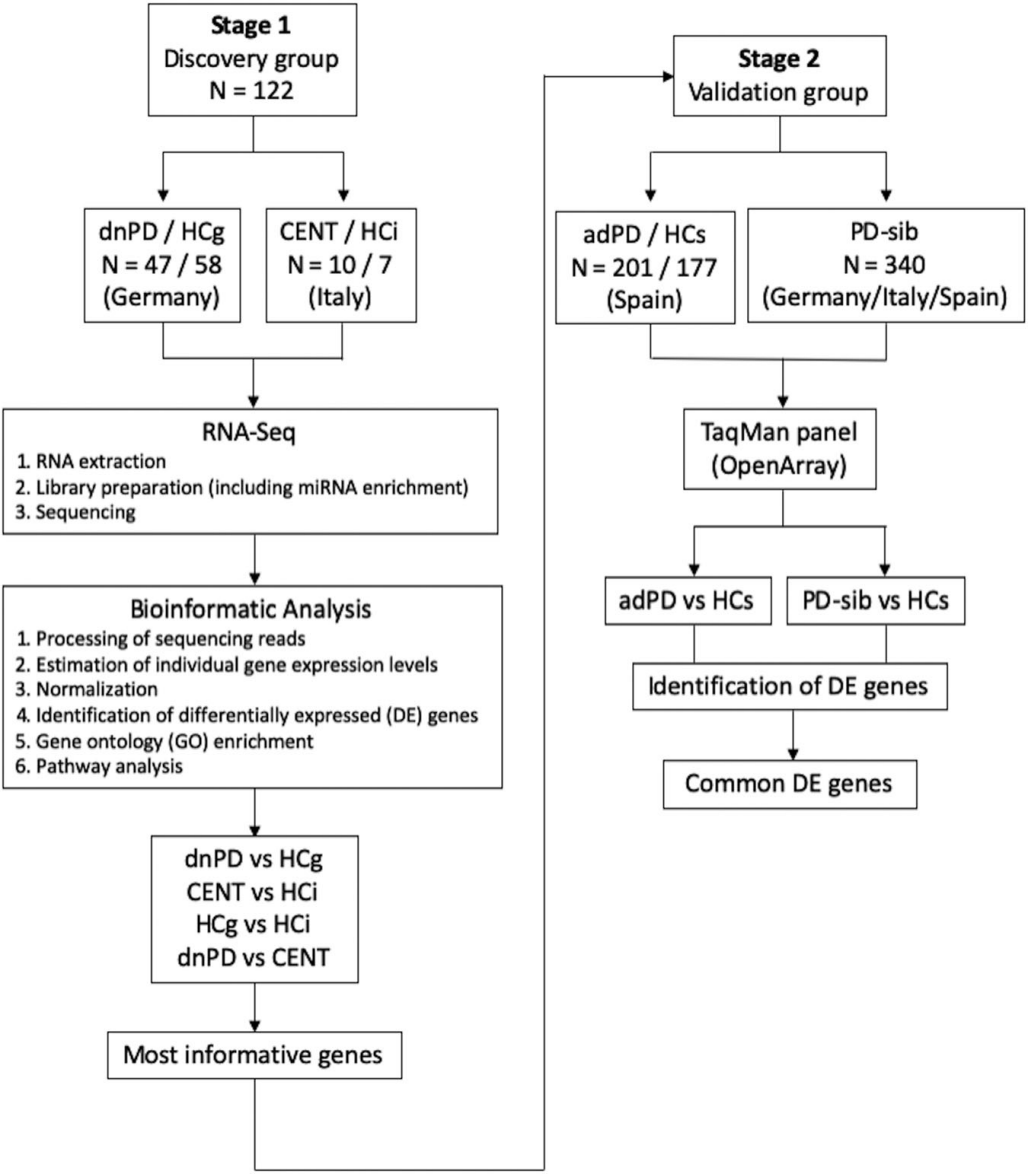
Multistage study design for discovery and validation of genes associated with Parkinson’s disease. N number of samples, dnPD de novo Parkinson’s disease, CENT centenarian, adPD advanced Parkinson’s disease, HCg healthy controls from Germany, HCi healthy controls from Italy, HCs healthy controls from Spain, PD Parkinson’s disease, PD-sib siblings of patients with Parkinson’s disease, DE differential expression.

We first investigated the gene expression profile to identify novel diagnostic biomarkers for PD within the framework of the aging process (Fig. 1). Therefore, two comparisons (dnPD vs. HCg, dnPD vs. CENT) were performed to ascertain the differences in the transcriptomic signatures between PD and longevity/successful aging. Two additional comparisons were performed to determine the differences associated with aging/longevity (CENT vs. HCi) or with ethnicity (HCg vs. HCi).

##### Differential gene expression analysis of dnPD vs. HCg

We conducted a principal component analysis (PCA) to evaluate the variance structure of our data, indicating a sex bias in the global gene expression profiles (Supplementary Fig. 1A). The highest percentage of variance explained by the first 2 components was low (36% variance).

Adjusting for sex, a total of 186 transcripts (including 7 long non-coding RNAs, 172 protein-coding RNAs, 5 pseudogenes, and 2 to be experimentally confirmed [TEC]) showed significant changes in transcript abundance after FDR correction. In the dnPD group, 44 transcripts were observed to be downregulated and 142 to be upregulated (Supplementary Fig. 1B). Supplementary Table 1 lists the top 10 upregulated and downregulated transcripts. The results of the GO analysis revealed that the differentially expressed genes (DEGs) between the dnPD and HCg groups were significantly enriched in pathways mostly related to immune function and inflammation (Supplementary Table 2).

##### Differential gene expression analysis of dnPD vs. CENT

Due to the fact that CENT never showed clinical signs of motor disability, they were considered “super-controls,” and a DGE analysis comparing the dnPD and CENT cohorts was performed. As shown in the previous approach, the PCA plot showed a sex bias in the global gene expression profiles (Supplementary Fig. 1C). Here, the highest percentage of variance explained by the first 2 components was also low (36% variance).

Therefore, the DGE analysis of the dnPD cohort (from Germany) compared with the CENT cohort (from Italy), adjusted by sex, identified 876 DEGs with an FDR of 0.05 (Supplementary Fig. 1D). Supplementary Table 1 lists the top 10 upregulated and downregulated transcripts. The results of the GO analysis are shown in Supplementary Table 3. The cohorts were from different countries, and therefore the DEGs identified could be due to the condition alone or due to the sample’s different ethnicity.

##### Differential gene expression analysis of HCg vs. HCi

Because genetic variation influences gene expression, a DGE analysis comparing the two HC cohorts (HCg and HCi) was therefore performed to clarify the aspect of sample’s different ethnicity, and found strong differences between the cohorts (Supplementary Table 1).

##### Differential gene expression analysis of CENT vs. HCi

To determine the genes related to longevity or aging, we also performed a DGE analysis of the CENT cohort compared with the HCi (both from Italy). We used sex as a confounding variable because, as shown in the previous approaches, the PCA plot showed a sex bias in the global gene expression profiles (Supplementary Fig. 1E). In this case, the highest percentage of variance explained by the first 2 components was higher (42% variance).

Adjusting for sex, a total of 304 DEGs (including 18 long non-coding RNAs, 275 protein-coding RNAs, 8 pseudogenes, 1 miRNA, 1 TEC, and 1 TR-C) were identified between these two groups (Supplementary Fig. 1F), with an FDR of 0.05. Supplementary Table 1 lists the top 10 upregulated and downregulated transcripts. The most prominent overrepresented pathway was the protein targeting the endoplasmic reticulum (Supplementary Table 4).

#### Identification of targets for validation

**Fig. 2:**
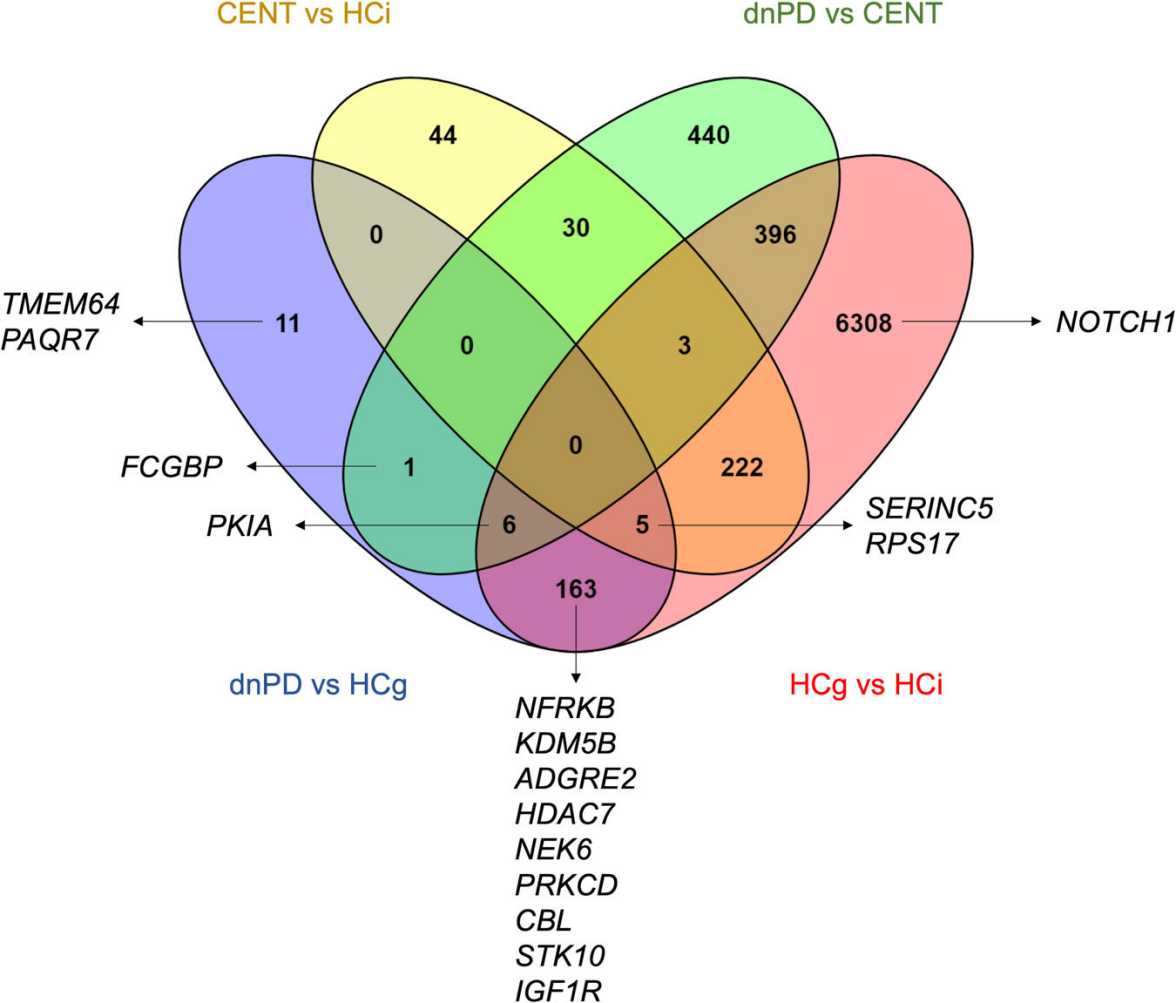
Venn diagram of differentially expressed genes per comparison made in the validation phase. The 16 targets selected for validation are shown (considering our results and those from other omic analyses in the context of the PROPAG-AGEING project).

The final targets selected for the validation stage were chosen based on the largest significant differential expression considering our four approaches and the intersection with the results from the PROPAG-AGEING project, a multiomic approach for the study of PD and age. Therefore, 16 genes were selected (Fig. 2).

### Validation stage

#### Differentially expressed genes in the validation cohorts via quantitative PCR

To validate the selected DEGs from the RNA-Seq analysis, we used two independent groups of samples (Fig. 1), including a total of 718 samples. Table 2 lists the participants’ demographic characteristics.

##### Differential gene expression analysis of patients with advanced Parkinson’s disease vs. healthy controls

A total of 378 samples, 201 adPDs and 177 HCs, all from Spain, were included in the analysis (Table 2).

**Fig. 3:**
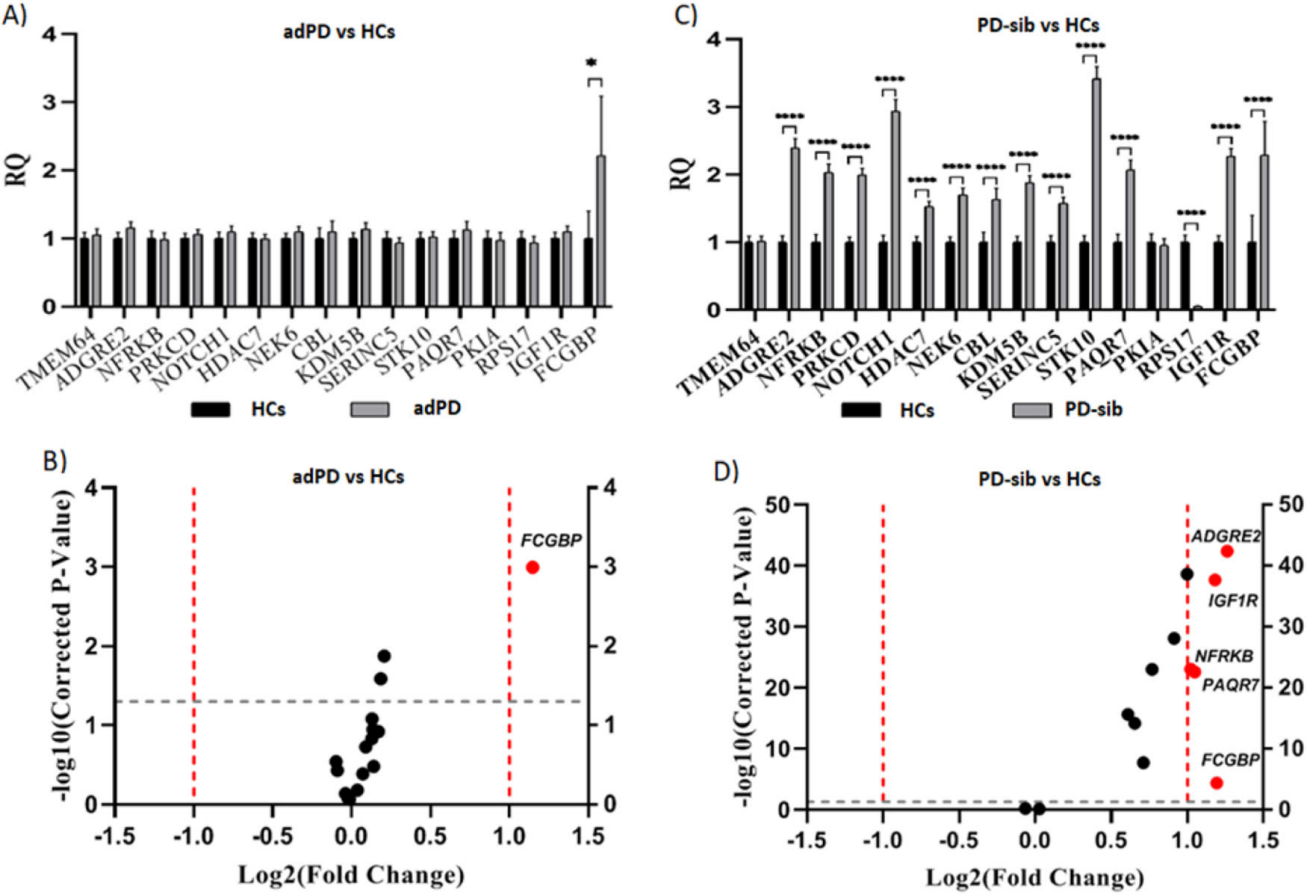
Analysis of differentially expressed genes in the validation cohorts. **A**, **C** qPCR analysis of the expression of those 16 genes selected from the discovery phase. *X*-axis, gene name; *Y*-axis, relative quantification (RQ) in gene expression. **B**, **D** Volcano plots of DEGs between groups. Red spot, upregulated; black spot, no difference in expression.

Expression changes of the 16 selected genes from the discovery stage were analyzed in adPDs compared with HCs by OpenArray® assays. The analysis revealed that *FCGBP* was the only DEG between the two cohorts (Fig. 3A, B and Supplementary Table 2).

##### Differential gene expression analysis of siblings of patients with sporadic Parkinson’s disease vs. healthy controls

We then performed the DGE analysis of PD-sibs compared with HCs. For this analysis, a total of 517 samples from 340 PD-sibs and 177 HCs were included (Table 2).

The results showed that the transcriptomic profile of the PD-sibs was comparable to that of the dnPD cohort, whereas the transcriptomic profile of the adPDs differed from that of the dnPDs (Fig. 3C, D and Supplementary Table 2). Consequently, only the *FCGBP* gene showed significant differences in dnPDs and adPDs and in those at risk of PD, such as PD-sibs.

## Discussion

This transcriptomic study included dnPD and adPD cases, CENTs, PD-sibs, and HCs. Given that aging is a high-risk factor in PD, we used the CENTs as “supercontrols.” We included PD-sibs because they have been reported to have a higher genetic risk of PD^8^. We found differential expression levels between dnPD cases and HCs, as well as between dnPD cases and CENTs. The differentially expressed genes included *FCGBP*, which was the only one that presented an altered expression in both comparisons and showed no differences in terms of population origin or aging process. We also showed altered *FCGBP* expression in the adPD cases and in a risk population such as PD-sibs. Interestingly, PD-sibs differed more than adPD from the HCs, probably pointing out to compensatory mechanisms associated with the development of the disease. Although it may not represent an initiating factor in all-case PD, chronic neuroinflammation appears to be a cofactor for disease progression^9^. To the best of our knowledge, this is the first study to demonstrate that *FCGBP* is related to PD from the initial stages in all populations.

Located on chromosome 19q13.2, *FCGBP* gene was upregulated in all our case-control comparisons. The gene encodes for the IgG Fc binding protein, and was first described with a putative important role in the immune protection and inflammation process in the intestines^10^. The gene’s expression has been reported to be strongly induced by the TH2 cytokine interleukin-13^11^. *FCGBP* is widely expressed on mucosal surfaces and in external secretions and is functionally intact in several fluids, lending support to the concept that *FCGBP* is an important component of mucosal immunological defenses^12^.

It has been suggested that the gut microbiota triggers mucosal immune activation, leading to neuroinflammation and neurodegeneration in PD^13^. The mucosal integrity of the entire gastrointestinal tract and its protection are vital for maintaining health. One of the most important factors involved in this protection is the integrity of the mucosal barrier, where the microbiome plays a major role^14^. In fact, the mucosal barrier is digested by the overgrowth of certain bacteria, such as *Akkermansia*, which has been observed to be more abundant in patients with PD^15^.

The gut microbiota has been implicated in the pathogenesis of several neurological disorders, such as PD, Alzheimer’s disease, multiple sclerosis, and amyotrophic lateral sclerosis. In recent years, studies have shown evidence of dysbiosis (an abnormal gut microbiota) in PD influenced by several factors, including dietary habits, chronic stress, exposure to toxins, and genetic background^15^. Evidence suggests that abnormalities in gut microbiota could contribute to neuroinflammation and motor progression of PD^16^.

Trefoil factor (TFF) peptides are a family of mucin-associated secretory molecules that play numerous physiological roles in maintaining and restoring gastrointestinal mucosal homeostasis and in response to gastrointestinal mucosal injury and inflammation. Although mucosal epithelia are the predominant TFF expression sites, TFF peptides appear in many body fluids^17^. Minute amounts of TFF peptides are also secreted in an endocrine manner. In particular, TFF3 is synthesized in neurons (such as the oxytocinergic neurons of the hypothalamus), activated microglial cells, and astrocytes of the brain. In fact, cerebral TFF3 has been reported to be involved in several processes, such as fear, depression, and learning. Furthermore, TFF3 has been linked with neurodegenerative and neuropsychiatric disorders^18,19^. Interestingly, *FCGBP* can be found forming disulfide-linked heterodimers with TFF1 and TFF3, at least in the intestines, with relative amounts of TFF3-FCGBP much higher than that of TFF1-FCGBP^20^.

The function of *FCGBP* is poorly understood; however, its characterization in differential expression analyses has revealed that this gene is involved in several disorders in which immune and inflammation processes are important in the onset and development of the disease. Thus, downregulation of this gene has been related to different types of cancer, suggesting a key role in homeostasis. In fact, it has been speculated that *FCGBP* could be a tumor suppressor gene^21,22^.

Recent researches have shown that FCGBP is not restricted to a gastrointestinal context; indeed it is a circulating biomarker. Therefore, it has been proved higher level of FCGBP in the blood stream of patients with autoimmune diseases, suggesting an increased generation of FCGBP in goblet cells and its secretion into the circulation by an unknown mechanism^23^. In addition, it has been described an inflammatory biomarker signature, including FCGBP, in small extracellular vesicles isolated from blood samples of glioblastoma patients^24^.

Brain arteriovenous malformation is a major cause of cerebral hemorrhage and is due to an abnormal connection between cerebrovascular arteries and veins. A recently published study on brain arteriovenous malformation using whole-exome sequencing in trios^25^ identified compound heterozygous variants that were recurrent in more than one trio in 16 genes, including *FCGBP*. More importantly, however, elevated *FCGBP* levels have been reported as contributing to the pathogenesis of several neurological disorders in which inflammation and intestinal dysbiosis have been involved. *FCGBP* has therefore been related to amyotrophic lateral sclerosis, a fatal disorder caused by the progressive degeneration of motoneurons in the brain and spinal cord, by facilitating autoimmune and neuroinflammatory responses^26^. A transcriptomic analysis of the dorsal striatum comparing individuals with bipolar disorder and controls found significant changes in the expression of 14 genes, including a few immune response genes, such as *NLRC5*, *S100A12*, *LILRA4*, and *FCGB*^27^. A study of patients with Friedreich’s ataxia found a group of differentially expressed transcripts. Interestingly, *FCGBP* was among the top 10 genes with the most differential expression between patients and controls and among the 13 transcripts significantly associated with disease duration^28^. A transcriptomic analysis of the hippocampal CA1 region was conducted in a cohort of patients with late onset Alzheimer’s disease (LOAD) and included patients with PD as “neuro-inflammatory disease” controls to identify LOAD-specific transcriptomic changes not shared with general neuro-inflammatory processes^29^. The study found 11 genes, including *FCGBP*, that were differentially expressed between the LOAD and control groups and between the hippocampal PD samples and control groups.

Although no prior studies have reported *FCGBP* as a biomarker for PD, there is a whole exome sequencing study that described a genetic variant (p.Glu465fs) in the *FCGBP* gene in 2 patients with PD but not in controls^30^.

Despite the novel results, there were limitations of the current study. The primary limitation is the potential genetic heterogeneity of the samples in both the discovery and replications cohorts from different European countries since there is no confirmation with genome-wide genotyping data. In addition, the selection of the 16 genes from RNA-Seq was based on ranking from the DEGs, which may bias the findings. On the other hand, although blood expression of *FCGBP* is altered in our study and this may be correlated with its transcriptional dysregulation in gut, it is important to remember two aspects, first *FCGBP* expression has been described not only in gut but also in several mucous epithelia of various organs^12^, and second, the expression levels do not always correlate with protein concentrations.

Altogether, our study suggests that FCGBP-mediated inflammation could have an important role in intestinal and brain inflammation, which could lead to neurodegeneration, the major cause of cognitive and motor dysfunction. A growing number of studies also support the involvement of both innate and adaptive immune responses in neurodegeneration. There is growing awareness that the immune system is inextricably involved not only in mediating damage but also in regenerating and repairing the damage in neurodegenerative disorders^31^. FCGBP-mediated inflammation can start very early within the progression of PD, leading to the concept that modifier interventions might be implemented even in the premotor phase of the disease.

Our results agree with the previously suggested idea that the PD process is systemic and not restricted to neurological tissues^4^. Moreover, a recent study provided further support for the importance of immune mechanisms in PD pathogenesis^32^.

The elevated expression of *FCGBP* may reflect pathophysiologic changes in the context of PD etiology and could be related with some non-motor signs present in the prodromal stage. *FCGBP* expression levels of the PD-sibs were comparable to that of the dnPD, highlighting a role from early-stage. However, it is necessary to keep in mind that it has been described that siblings show a heterogeneous distribution of prodromal PD markers and probability^4^. Therefore, the precise role of FCGBP in early-stage diagnosis and in prodromal stage requires further study.

Finally, altered expression of FCGBP has been described in several disorders related with inflammation and immunologic response; due to the complexity of these processes the use of *FCGBP* as diagnosis biomarker is still ambiguous. Additional studies, such as genetic or proteomic studies, will help us understand its relevance in PD and to establish whether FCGBP may be used as a marker for the early diagnosis and/or prognosis of disease processes specifically for PD.

In summary, we provide evidence for upregulation of *FCGBP* mRNA levels, not only in patients with PD but also in those with a high risk of PD, suggesting that *FCGBP* could be important in PD gut-brain interactions, mediating the connection between the microbiota and intestinal inflammatory processes, as well as in neuroinflammation and neurodegeneration. Further studies are required to confirm our findings.

## Methods

### Participants and study design

This study included participants from the multicenter PROPAG-AGEING project^33^ in both the discovery and validation stages (Fig. 1). All participants were self-identified to be of European ancestry, they were older than 18 years of age, and they were extensively phenotyped from the clinical point of view. PD was diagnosed by movement disorder neurologists according to the United Kingdom Parkinson’s Disease Society Brain Bank criteria^34^. The healthy controls (HCs), healthy centenarians (CENT), and siblings of patients with a diagnosis of sporadic PD (PD-sibs) had no active known/treated central nervous system condition, as determined during clinical interview. The PD-sibs cohort was deeply characterized for several clinical parameters, with particular regard for motor and non-motor symptoms and video-polysomnography-confirmed REM sleep behavior disorder^7^. Accurate evaluation of these parameters allowed estimating the risk of developing PD. After the clinical diagnosis, peripheral blood samples were collected from each participant since peripheral whole blood is easily accessible and has low risk associated with its collection, as compared to other more invasive procedures such as CSF collection, making it ideally suited to the development of diagnostic biomarker tests.

PD-sibs were recruited over 20 months, between September 2016 and January 2019, by the Local Health Unit of Bologna—Institute of Neurological Sciences of Bologna (Italy), Andalusian Health Service (Spain), and Paracelsus Elena Clinic Kassel/University Medical Center Göttingen (Germany).

For the discovery stage, a total of 122 participants from Germany and Italy were included. Therefore, we analyzed 47 patients with de novo PD (dnPD; those with no history of present or past therapy with anti-Parkinsonian drugs, from the longitudinal de novo Parkinson cohort^35^), 58 HCs from Germany and 7 from Italy (HCg and HCi, respectively), and 10 CENTs. Table 1 lists the participants’ demographic characteristics.

**Table 1:** Demographic characteristics of the discovery cohorts.

Origin	Germany		Italy	
Phenotype	dnPD	HCg	CENT	HCi
*N*	47	58	10	7
Sex, M/F (M %)	34/13 (72.3)	33/25 (56.9)	2/8 (20.0)	4/3 (57.1)
Age ± SD, years	70 ± 9. 2	70.9 ± 6.7	103.8 ± 2.9	68.4 ± 8.0
AO ± SD, years	62.0 ± 9.1	–	–	–

*N* number of participants, *M* male, *F* female, *SD* standard deviation, *Y* years, *dnPD* de novo Parkinson’s disease, *HC* healthy control, *CENT* centenarians (healthy “supercontrols”), *AO* age at onset, *SD* standard deviation.

For the second stage of validation, we established 3 independent cohorts: 201 patients with advanced PD (adPD; disease duration of at least 5 years) (2.5% patients carried pathogenic *LRRK2* and *PRKN* mutations), 177 HCs, and 340 PD-sibs from Spain, Germany, and Italy. Table 2 summarizes the participants’ demographic characteristics.

**Table 2:** Demographic characteristics of the validation cohorts.

Phenotype	adPD	HCs	Siblings of patients with PD		
Origin	Spain		Spain	Italy	Germany
*N*	201	177	120	100	120
Sex, M/F (M %)	120/81 (59.7)	94/83 (53.1)	51/69 (42.5)	44/56 (44)	46/74 (38.3)
Age ± SD, years	63.4 ± 11.0	53.5 ± 14.8	57.8 ± 11.2	63.6 ± 9.6	65.2 ± 9.7
AO ± SD, years	51.5 ± 13.0	–	–	–	–
YE ± SD, years	10.4 ± 7.4	–	–	–	–

*N* number of participants, *M* male, *F* female, *SD* standard deviation, *Y* years, *adPD* advanced Parkinson’s disease, *HC* healthy control, *AO* age at onset, *YE* years of evolution.

### Ethics statements

The study was approved by the local ethics committees of all centers participating in the consortium (UMG-GOE ethics committee approval no. 19/5/16 of August 2016, ISNB ethics committee approval no. 16018 of May 2016, SAS ethical committee approval no. 2014/PI173 of September 2016) and was conducted according to the principles expressed in the Declaration of Helsinki. Each study participant signed a written informed consent document before undergoing blood draws.

### RNA isolation

We extracted total RNA from whole blood samples using the PAXgene Blood miRNA Kit (PreAnalytix, Qiagen, Hilden, Germany), following the manufacturer’s protocol. We quantified the isolated RNA using a ND-1000 spectrophotometer (NanoDrop Technologies, Wilmington, DE, USA) and removed DNA from the samples using RNase-free DNase (Promega, Madison, WI, USA), also according to the manufacturer’s instructions. We checked the integrity of the RNA with an Agilent 2100 Bioanalyzer, in conjunction with the RNA 6000 Nano kit (Agilent Technologies, CA, USA). RNA integrity number (RIN) value of the samples was of 7.0 or higher.

### RNA-Seq library construction and sequencing

We constructed the RNA libraries using the TruSeq^TM^ stranded total RNA kit with Ribo-Zero (Illumina, San Diego, CA, USA), with equal quantities of high-quality RNA from each sample and following the manufacturer’s instructions. We tested the quality and amount of total RNA with TapeStation 4200 and Qubit 3.0, pooling and quantifying the libraries by reverse transcription-polymerase chain reaction, and subsequently sequenced the RNA in an Illumina HiSeq 25000 (2 × 101 pb; 40 M reads/sample; *Q* ≥ 30 ≥ 90%).

### Data analysis

We aligned the sequence files against the hg38 reference genome with TopHat v.2.0.6 to take into account exon–exon splice junctions. We excluded reads that had not mapped uniquely to a genome position, obtaining the number of reads at the gene level with HTSeq software v.0.6.0. We analyzed the resulting counts with the DESeq2 Bioconductor package in R v.3.6.0, which estimates size factors based on the median-of-ratios method, fit dispersions, and performs the differential expression analysis using negative binomial generalized linear models. Sex was included as covariate in the design formula, whereas other experimental covariates (such as RIN or age) were not considered since no differences were detected between groups. We performed multiple testing correction adjustment employing the false discovery rate (FDR) method (FDR p-value threshold of 0.05). For functional annotation of gene ontology (GO), we used the web-based DAVID v.6.8 tool.

### Validation of RNA-Seq by qPCR

We performed the validation of the DGE findings from the RNA-Seq analysis on a QuantStudioTM 12 K Flex OpenArray® Real-Time PCR System (Thermo Fisher Scientific, CA, USA).

We extracted RNA employing the automated RNA extractor MAxwell® 16 System (Promega) according to the manufacturer’s protocol and reverse transcribed the total RNA with random hexamers using the High-Capacity cDNA Reverse Transcription Kit (Applied Biosystems) according to the manufacturer’s instructions.

The OpenArray panel was custom designed and included the 16 candidate genes selected from the discovery stage. We also added 2 reference (housekeeping) genes, *ACTB* and *UBC*, for qPCR data normalization. Supplementary Table 2 lists the accession numbers for the genes analyzed by OpenArray. For each candidate gene, a TaqMan® assay was custom designed by the Bioinformatics Group at Life Technologies and distributed in the OpenArray 384-well sample plates, each containing 48 subarrays. To minimize the technical variability, OpenArray was performed in triplicate on each sample. Additionally, samples were randomized on the OpenArray plates to minimize batch effects. We used standard cycling conditions as recommended by the manufacturer. Normalized expression levels for each experimental data point were calculated using the reference genes. We performed the data analysis using the Thermo Fisher Cloud v.1.0 platform and based the selection of significant genes on FC≥|2| and corrected *p* values <0.05.

### Reporting summary

Further information on research design is available in the Nature Research Reporting Summary linked to this article.

## Supplementary information

Supplementary Material

Reporting Summary

## Data Availability

The RNA-Seq count data that support the findings of this study are available on request from the corresponding author [P.M., P.G.-G.].
