# Supplementary material for: Transcriptomic analysis reveals an association of FCGBP with Parkinson’s disease
Source: NPJ Parkinsons Dis. 2022 Nov 12;8:157. doi: 10.1038/s41531-022-00415-7 (PMC9653420; doi:10.1038/s41531-022-00415-7)
Supplement: Supplementary file 1 — Supplementary Material [file 41531_2022_415_MOESM1_ESM.pdf]

SUPPLEMENTARY MATERIAL

Supplementary Figure 1. Principal component analysis and volcano plots of differentially expressed transcripts from the different comparisons. (A, C, E) PCA plots showing normalized counts, colored by phenotypic group and sex. (B, D, F) Volcano plots showing differentially expressed genes (DEGs). Log2-fold change was plotted against the DESeq2 R-generated p-value (-log base 10). Red spots represent significant DEGs. Differential expression analysis using an adjusted p-value < 0.05 cutoff identified 186 DEGs between dnPD and HCg, 876 between dnPD and CENT, and 304 between CENT and HCi. F, female; M, male; dnPD, de novo Parkinson's disease (from Germany); DEG, differentially expressed gene; HCg, healthy controls from Germany; CENT, centenarian (from Italy); HCi, healthy controls from Italy; PCA, principal component analysis.

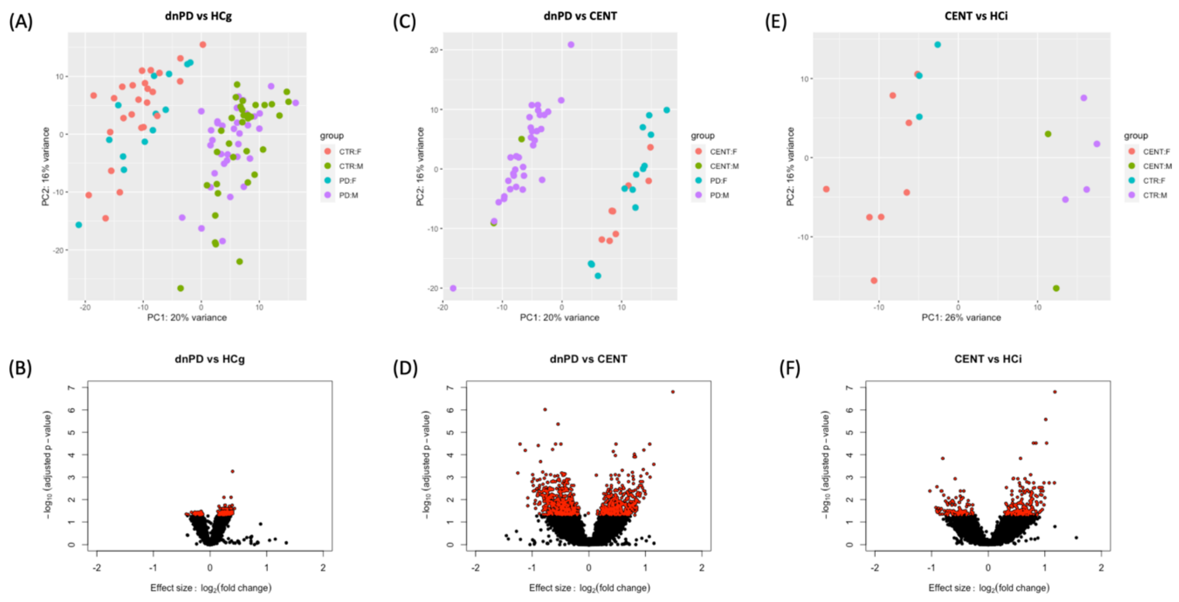

Supplementary Table 1. List of top 10 upregulated/downregulated transcripts in the different approaches.

Comparison		Gene type	ensembl_gene_ID/transcript ID	hgnc_symbol	Name	entrezgene	log2FoldChange	p-value	p-value$_{adj}$
dnPD vs HCg	Higher in dnPD	Protein-coding	ENSG00000119408	NEK6	NIMA related kinase 6	10783	0.3960	4.79E-08	5.54E-04
		Protein-coding	ENSG00000128815	WDFY4	WDFY family member 4	57705	0.3705	1.37E-06	7.93E-03
		Protein-coding	ENSG00000117139	KDM5B	Lysine demethylase 5B	10765	0.2440	2.06E-06	7.96E-03
		Protein-coding	ENSG00000127507	ADGRE2	Adhesion G protein-coupled receptor E2	30817	0.3899	9.20E-06	1.79E-02
		Protein-coding	ENSG00000124422	USP22	Ubiquitin specific peptidase 22	23326	0.2683	9.28E-06	1.79E-02
		Protein-coding	ENSG00000170322	NFRKB	Nuclear factor related to kappaB binding protein	4798	0.2601	6.77E-06	1.79E-02
		Protein-coding	ENSG00000156711	MAPK13	Mitogen-activated protein kinase 13	5603	0.3413	1.36E-05	1.97E-02
		Protein-coding	ENSG00000047056	WDR37	WD repeat domain 37	22884	0.1576	1.20E-05	1.97E-02
		Protein-coding	ENSG00000168916	ZNF608	Zinc finger protein 608	57507	0.4310	3.85E-05	2.49E-02
		Protein-coding	ENSG00000281106	TMEM272	Transmembrane protein 272	283521	0.3799	3.83E-05	2.49E-02
	Lower in dnPD	Pseudogene	ENSG00000163875	MEAF6	MYST/Esa1-associated factor 6	64769	-0.1588	9.66E-05	3.31E-02
		Protein-coding	ENSG00000142875	PRKACB	Protein kinase cAMP-activated catalytic subunit beta	5567	-0.2703	1.40E-04	3.59E-02
		Protein-coding	ENSG00000173960	UBXN2A	UBX domain protein 2A	165324	-0.1690	1.48E-04	3.59E-02
		Protein-coding	ENSG00000064886	CHI3L2	Chitinase 3 like 2	1117	-0.3278	1.71E-04	3.66E-02
		Protein-coding	ENSG00000179476	C14orf28	Chromosome 14 open reading frame 28	122525	-0.2602	1.78E-04	3.66E-02
		Protein-coding	ENSG00000117155	SSX2IP	SSX family member 2 interacting protein	117178	-0.2566	2.69E-04	3.66E-02
		Protein-coding	ENSG00000167904	TMEM68	Transmembrane protein 68	137695	-0.2183	2.62E-04	3.66E-02
		Protein-coding	ENSG00000080839	RBL1	RB transcriptional corepressor like 1	5933	-0.1912	2.68E-04	3.66E-02
		Protein-coding	ENSG00000163807	KIAA1143	KIAA1143	57456	-0.1693	2.09E-04	3.66E-02
		Protein-coding	ENSG00000160049	DFFA	DNA fragmentation factor subunit alpha	1676	-0.1503	2.19E-04	3.66E-02
dnPD vs CENT	Higher in dnPD	Protein-coding	ENSG00000173114	LRRN3	Leucine rich repeat neuronal 3	54674	1.4855	7.53E-12	1.57E-07
		Protein-coding	ENSG00000104660	LEPROTL1	Leptin receptor overlapping transcript like 1	23484	0.4794	9.37E-09	3.35E-05
		Protein-coding	ENSG00000135318	NT5E	5,-nucleotidase ecto	4907	1.0764	1.28E-08	3.35E-05
		Protein-coding	ENSG00000278195	SSTR3	Somatostatin receptor 3	6753	0.9715	3.25E-08	6.16E-05
		Protein-coding	ENSG00000163684	RPP14	Ribonuclease P/MRP subunit p14	11102	0.4315	4.46E-08	7.16E-05
		Protein-coding	ENSG00000154153	RETREG1	Reticulophagy regulator 1	54463	0.8041	6.39E-08	9.52E-05
		Protein-coding	ENSG00000181896	ZNF101	Zinc finger protein 101	94039	0.4691	7.80E-08	1.08E-04
		Protein-coding	ENSG00000165272	AQP3	Aquaporin 3	360	0.7993	1.01E-07	1.24E-04
		Protein-coding	ENSG00000185875	THNSL1	Threonine synthase like 1	79896	0.8227	9.93E-08	1.24E-04
		Protein-coding	ENSG00000183691	NOG	Noggin	9241	1.1484	2.44E-07	2.68E-04
	Lower in dnPD	LncRNA	ENSG00000222041	CYTOR	Cytoskeleton regulator RNA	112597	-0.7718	9.27E-11	9.67E-07
		Protein-coding	ENSG00000144136	SLC20A1	Solute carrier family 20 member 1	6574	-0.5431	6.24E-10	4.34E-06
		Protein-coding	ENSG00000160219	GAB3	GRB2 associated binding protein 3	139716	-0.4942	9.83E-09	3.35E-05
		Protein-coding	ENSG00000169554	ZEB2	Zinc finger E-box binding homeobox 2	9839	-0.5861	1.29E-08	3.35E-05
		Protein-coding	ENSG00000183091	NEB	Nebulin	4703	-1.2146	9.63E-09	3.35E-05
		TEC	ENSG00000279166	AC009951.4	Novel transcript	.	-0.8919	1.67E-08	3.87E-05
		LncRNA	ENSG00000261438	AL157394.1	Novel transcript	.	-0.6389	2.78E-08	5.80E-05
		LncRNA	ENSG00000235531	MSC-AS1	MSC antisense RNA 1	100132891	-1.1216	3.68E-08	6.40E-05
		Protein-coding	ENSG00000198846	TOX	Thymocyte selection associated high mobility group box	9760	-0.8524	1.75E-07	2.03E-04
		Protein-coding	ENSG00000086288	NME8	NME/NM23 family member 8	51314	-0.8141	6.21E-07	5.89E-04
CENT vs Hci	Higher in CENT	Protein-coding	ENSG00000229117	RPL41	Ribosomal protein L41	6171	1.1790	1.16E-11	1.58E-07
		Protein-coding	ENSG00000122026	RPL21	Ribosomal protein L21	6144	1.0172	3.94E-10	2.68E-06
		Protein-coding	ENSG00000123349	PFDN5	Prefoldin subunit 5	5204	0.8441	8.03E-09	3.03E-05
		Protein-coding	ENSG00000135046	ANXA1	Annexin A1	301	0.7980	9.44E-09	3.03E-05
		LncRNA	ENSG00000278771	RN7SL3	RNA component of signal recognition particle 7SL3	378707	1.0311	1.11E-08	3.03E-05
		Protein-coding	ENSG00000115339	GALNT3	Polypeptide N-acetylgalactosaminyltransferase 3	2591	0.5715	7.53E-08	1.46E-04
		Protein-coding	ENSG00000156482	RPL30	Ribosomal protein L30	6156	0.7490	4.58E-07	7.79E-04
		Protein-coding	ENSG00000120265	PCMT1	Protein-L-isoaspartate (D-aspartate) O-methyltransferase	5110	0.4460	7.46E-07	1.13E-03
		Protein-coding	ENSG00000109270	LAMTOR3	Late endosomal/lysosomal adaptor, MAPK and MTOR activator 3	8649	0.5248	1.02E-06	1.18E-03
		Protein-coding	ENSG00000156467	UQCRB	Ubiquinol-cytochrome c reductase binding protein	7381	0.6491	1.13E-06	1.18E-03
	Lower in CENT	Protein-coding	ENSG00000010610	CD4	CD4 molecule	920	-0.8038	6.47E-08	1.46E-04
		Protein-coding	ENSG00000204351	SKIV2L	Ski2 like RNA helicase	6499	-0.5850	1.07E-06	1.18E-03
		Protein-coding	ENSG00000117643	MAN1C1	Mannosidase alpha class 1C member 1	57134	-0.9062	4.35E-06	2.69E-03
		Protein-coding	ENSG00000100401	RANGAP1	Ran GTPase activating protein 1	5905	-0.4920	1.21E-05	4.14E-03
		Protein-coding	ENSG00000112182	BACH2	BTB domain and CNC homolog 2	60468	-0.8307	1.18E-05	4.14E-03
		Protein-coding	ENSG00000168612	ZSWIM1	Zinc finger SWIM-type containing 1	90204	-0.8210	1.05E-05	4.14E-03
		Protein-coding	ENSG00000204371	EHMT2	Euchromatic histone lysine methyltransferase 2	10919	-0.4314	1.12E-05	4.14E-03
		Protein-coding	ENSG00000242498	ARPIN	Actin related protein 2/3 complex inhibitor	348110	-1.0304	1.22E-05	4.14E-03
		Protein-coding	ENSG00000079435	LIPE	Lipase E, hormone sensitive type	3991	-0.7872	2.41E-05	6.32E-03
		Protein-coding	ENSG00000101347	SAMHD1	SAM and HD domain containing deoxynucleoside triphosphate triphosphohydrolase 1	25939	-0.4962	2.86E-05	6.96E-03
HCg vs Hci	Higher in HCg	Protein-coding	ENSG00000121774	KHDRBS1	KH RNA binding domain containing, signal transduction associated 1	10657	-0.75203982	4.22E-16	1.11E-11
		Protein-coding	ENSG00000128815	WDFY4	WD repeat-and FYVE domain-containing protein 4	57705	-1.077272209	2.76E-13	3.64E-09
		Protein-coding	ENSG00000174227	PIGG	Phosphatidylinositol glycan anchor biosynthesis class G	54872	-0.585138161	1.71E-12	1.51E-08
		Protein-coding	ENSG00000172661	WASHC2C	WASH complex subunit 2C	253725	-0.766773864	4.57E-12	2.41E-08
		LncRNA	ENSG00000278231	-	Novel transcript	-	-1.201775042	6.67E-12	2.93E-08
		Protein-coding	ENSG00000180957	PITPNB	Phosphatidylinositol transfer protein beta	23760	-1.032895767	3.29E-11	8.67E-08
		Protein-coding	ENSG00000066136	NFYC	Nuclear transcription factor Y subunit gamma	4802	-0.678870152	8.45E-11	1.86E-07
		TEC	ENSG00000280138	AC027290.2	Novel transcript	-	-1.031486679	1.35E-10	2.74E-07
		Protein-coding	ENSG00000107929	LARP4B	La ribonucleoprotein 4B	23185	-0.825854317	2.37E-10	3.91E-07
		Protein-coding	ENSG00000131323	TRAF3	TNF receptor associated factor 3	7187	-0.590775163	2.72E-10	4.23E-07
	Lower in HCg	LncRNA	ENSG00000259001	-	Ribonuclease P RNA component H1	-	1.864668064	2.64E-12	1.75E-08
		Protein-coding	ENSG00000198346	ZNF813	Zinc finger protein 813	126017	1.128066039	9.14E-12	3.45E-08
		Protein-coding	ENSG00000170540	ARL6IP1	ADP ribosylation factor like GTPase 6 interacting protein 1	23204	0.619015013	1.37E-11	4.53E-08
		Protein-coding	ENSG00000187514	PTMA	Prothymosin alpha	5757	0.820036357	3.26E-11	8.67E-08
		Protein-coding	ENSG00000181472	ZBTB2	Zinc finger and BTB domain containing 2	57621	0.484783615	4.61E-11	1.11E-07
		Protein-coding	ENSG00000178700	DHFR2	Dihydrofolate reductase 2	200895	0.719757568	1.71E-10	3.22E-07
		Protein-coding	ENSG00000204186	ZDBF2	Zinc finger, DBF-type containing 2	57683	1.150928966	2.11E-10	3.72E-07
		Protein-coding	ENSG00000196267	ZNF836	Zinc finger protein 836	162962	0.792889255	3.17E-10	4.60E-07
		Protein-coding	ENSG00000205413	SAMD9	Sterile alpha motif domain containing 9	54809	1.217840969	4.61E-10	5.80E-07
		Protein-coding	ENSG00000213799	ZNF845	Zinc finger protein 845	91664	1.269238501	5.41E-10	6.49E-07

dnPD, *de novo* Parkinson's disease; HCg, healthy controls from Germany; HCi, healthy controls from Italy; CENT, centenarian (healthy "super-controls"); TEC, to be experimentally confirmed (non-spliced expressed sequence tag clusters that have polyA features); LncRNA, long non-coding RNA.

Supplementary Table 2. Gene ontology (GO) terms of DEGs between dnPD and HCg from DAVID.

Category	Term	Count	%	p-value	FDR
GOTERM_BP_ALL	myeloid cell activation involved in immune response	22	12.8	3.9E-9	1.3E-5
GOTERM_BP_ALL	myeloid leukocyte activation	23	13.4	1.9E-8	2.8E-5
GOTERM_BP_ALL	myeloid leukocyte mediated immunity	21	12.2	3.0E-8	2.8E-5
GOTERM_BP_ALL	cellular component organization or biogenesis	94	54.7	3.3E-8	2.8E-5
GOTERM_BP_ALL	leukocyte activation	33	19.2	6.2E-8	3.7E-5
GOTERM_BP_ALL	leukocyte degranulation	20	11.6	7.5E-8	3.7E-5
GOTERM_BP_ALL	cellular component organization	91	52.9	9.1E-8	3.7E-5
GOTERM_BP_ALL	leukocyte activation involved in immune response	23	13.4	9.9E-8	3.7E-5
GOTERM_BP_ALL	vesicle-mediated transport	42	24.4	1.1E-7	3.7E-5
GOTERM_BP_ALL	cell activation involved in immune response	23	13.4	1.1E-7	3.7E-5
GOTERM_BP_ALL	cell activation	34	19.8	2.5E-7	7.7E-5
GOTERM_BP_ALL	regulated exocytosis	22	12.8	1.8E-6	5.1E-4
GOTERM_BP_ALL	neutrophil activation involved in immune response	17	9.9	2.1E-6	5.5E-4
GOTERM_BP_ALL	neutrophil mediated immunity	17	9.9	2.9E-6	7.1E-4
GOTERM_BP_ALL	neutrophil activation	17	9.9	3.3E-6	7.1E-4
GOTERM_BP_ALL	positive regulation of biological process	83	48.3	3.3E-6	7.1E-4
GOTERM_BP_ALL	granulocyte activation	17	9.9	3.8E-6	7.5E-4
GOTERM_BP_ALL	immune system process	53	30.8	6.6E-6	1.2E-3
GOTERM_BP_ALL	regulation of Ras protein signal transduction	11	6.4	6.7E-6	1.2E-3
GOTERM_BP_ALL	neutrophil degranulation	16	9.3	8.3E-6	1.4E-3
GOTERM_BP_ALL	positive regulation of cellular process	75	43.6	9.7E-6	1.6E-3
GOTERM_BP_ALL	exocytosis	22	12.8	1.4E-5	2.1E-3
GOTERM_BP_ALL	small GTPase mediated signal transduction	15	8.7	1.9E-5	2.8E-3
GOTERM_BP_ALL	regulation of small GTPase mediated signal transduction	13	7.6	2.7E-5	3.8E-3
GOTERM_BP_ALL	protein metabolic process	80	46.5	2.8E-5	3.8E-3
GOTERM_BP_ALL	Ras protein signal transduction	13	7.6	4.0E-5	5.2E-3
GOTERM_BP_ALL	cellular protein modification process	58	33.7	5.6E-5	6.8E-3
GOTERM_BP_ALL	protein modification process	58	33.7	5.6E-5	6.8E-3
GOTERM_BP_ALL	histone modification	15	8.7	6.0E-5	7.0E-3
GOTERM_BP_ALL	cellular protein metabolic process	73	42.4	6.4E-5	7.1E-3
GOTERM_BP_ALL	macromolecule modification	60	34.9	6.5E-5	7.1E-3
GOTERM_BP_ALL	organelle organization	57	33.1	6.8E-5	7.2E-3
GOTERM_BP_ALL	establishment of cell polarity	8	4.7	7.4E-5	7.5E-3
GOTERM_BP_ALL	leukocyte mediated immunity	21	12.2	7.6E-5	7.5E-3
GOTERM_BP_ALL	covalent chromatin modification	15	8.7	7.8E-5	7.5E-3

GOTERM_BP_ALL	chromatin organization	20	11.6	8.6E-5	8.1E-3
GOTERM_BP_ALL	cellular macromolecule metabolic process	106	61.6	1.0E-4	9.3E-3
GOTERM_BP_ALL	establishment of T cell polarity	4	2.3	1.1E-4	9.4E-3
GOTERM_BP_ALL	establishment of localization in cell	36	20.9	1.1E-4	9.7E-3
GOTERM_BP_ALL	positive regulation of macromolecule metabolic process	49	28.5	1.3E-4	1.1E-2
GOTERM_BP_ALL	membrane organization	24	14.0	1.3E-4	1.1E-2
GOTERM_BP_ALL	establishment of lymphocyte polarity	4	2.3	1.4E-4	1.1E-2
GOTERM_BP_ALL	regulation of macromolecule metabolic process	81	47.1	1.6E-4	1.3E-2
GOTERM_BP_ALL	macromolecule metabolic process	111	64.5	2.0E-4	1.5E-2
GOTERM_BP_ALL	secretion by cell	25	14.5	2.7E-4	2.0E-2
GOTERM_BP_ALL	positive regulation of metabolic process	51	29.7	2.7E-4	2.0E-2
GOTERM_BP_ALL	regulation of localization	42	24.4	2.9E-4	2.1E-2
GOTERM_BP_ALL	regulation of establishment of T cell polarity	3	1.7	3.8E-4	2.6E-2
GOTERM_BP_ALL	immune effector process	25	14.5	3.8E-4	2.6E-2
GOTERM_BP_ALL	chromosome organization	24	14.0	4.0E-4	2.7E-2
GOTERM_BP_ALL	leukocyte migration	14	8.1	4.5E-4	3.0E-2
GOTERM_BP_ALL	cellular localization	42	24.4	4.8E-4	3.1E-2
GOTERM_BP_ALL	secretion	26	15.1	4.9E-4	3.1E-2
GOTERM_BP_ALL	intracellular transport	30	17.4	5.4E-4	3.4E-2
GOTERM_BP_ALL	regulation of metabolic process	84	48.8	5.6E-4	3.4E-2
GOTERM_BP_ALL	establishment of localization	64	37.2	7.0E-4	4.2E-2
GOTERM_BP_ALL	cellular metabolic process	119	69.2	7.3E-4	4.3E-2
GOTERM_BP_ALL	single-organism transport	45	26.2	7.7E-4	4.5E-2
GOTERM_BP_ALL	mast cell activation involved in immune response	5	2.9	8.5E-4	4.9E-2
GOTERM_BP_ALL	histone deacetylation	6	3.5	8.7E-4	4.9E-2

Supplementary Table 3. Gene ontology (GO) terms of DEGs between dnPD and CENT from DAVID.

Category	Term	Count	%	p-value	FDR
GOTERM_BP_ALL	regulation of transcription from RNA polymerase II promoter	143	19.3	2.6E-9	1.6E-5
GOTERM_BP_ALL	transcription, DNA-templated	172	23.2	9.4E-8	2.4E-4
GOTERM_BP_ALL	regulation of RNA biosynthetic process	171	23.0	2.3E-7	2.4E-4
GOTERM_BP_ALL	regulation of transcription, DNA-templated	170	22.9	2.3E-7	2.4E-4
GOTERM_BP_ALL	regulation of RNA metabolic process	178	24.0	2.4E-7	2.4E-4
GOTERM_BP_ALL	regulation of nucleic acid-templated transcription	170	22.9	2.4E-7	2.4E-4
GOTERM_BP_ALL	regulation of nucleobase-containing compound metabolic process	191	25.7	4.6E-7	3.9E-4
GOTERM_BP_ALL	nucleic acid-templated transcription	174	23.5	7.0E-7	5.2E-4
GOTERM_BP_ALL	regulation of metabolic process	302	40.7	1.1E-6	7.5E-4
GOTERM_BP_ALL	regulation of cellular metabolic process	280	37.7	1.6E-6	9.5E-4
GOTERM_BP_ALL	RNA biosynthetic process	176	23.7	2.1E-6	1.1E-3
GOTERM_BP_ALL	biological regulation	475	64.0	5.3E-6	2.7E-3
GOTERM_BP_ALL	heterocycle biosynthetic process	190	25.6	8.9E-6	3.8E-3
GOTERM_BP_ALL	regulation of macromolecule metabolic process	277	37.3	8.9E-6	3.8E-3
GOTERM_BP_ALL	nucleobase-containing compound biosynthetic process	187	25.2	1.0E-5	3.8E-3
GOTERM_BP_ALL	aromatic compound biosynthetic process	190	25.6	1.0E-5	3.8E-3
GOTERM_BP_ALL	regulation of biological process	452	60.9	1.2E-5	4.1E-3
GOTERM_BP_ALL	organic cyclic compound biosynthetic process	195	26.3	1.2E-5	4.1E-3
GOTERM_BP_ALL	regulation of gene expression	221	29.8	1.4E-5	4.3E-3
GOTERM_BP_ALL	regulation of primary metabolic process	271	36.5	1.8E-5	5.3E-3
GOTERM_BP_ALL	regulation of macromolecule biosynthetic process	198	26.7	2.6E-5	7.5E-3
GOTERM_BP_ALL	regulation of cellular process	427	57.5	4.5E-5	1.2E-2
GOTERM_BP_ALL	regulation of cellular macromolecule biosynthetic process	195	26.3	4.5E-5	1.2E-2
GOTERM_BP_ALL	RNA metabolic process	216	29.1	5.7E-5	1.4E-2
GOTERM_BP_ALL	regulation of nitrogen compound metabolic process	209	28.2	7.1E-5	1.7E-2
GOTERM_BP_ALL	regulation of biosynthetic process	204	27.5	8.6E-5	1.9E-2
GOTERM_BP_ALL	regulation of cellular biosynthetic process	202	27.2	8.7E-5	1.9E-2
GOTERM_BP_ALL	nucleic acid metabolic process	232	31.3	1.4E-4	3.0E-2

Supplementary Table 4. Gene ontology (GO) terms of DEGs between CENT and HCi from DAVID.

Category	Term	Count	%	p-value	FDR
GOTERM_BP_ALL	cotranslational protein targeting to membrane	28	9.8	2.5E-26	1.1E-22
GOTERM_BP_ALL	SRP-dependent cotranslational protein targeting to membrane	27	9.5	1.4E-25	3.0E-22
GOTERM_BP_ALL	protein targeting to ER	27	9.5	1.3E-23	2.0E-20
GOTERM_BP_ALL	establishment of protein localization to endoplasmic reticulum	27	9.5	3.2E-23	3.5E-20
GOTERM_BP_ALL	viral transcription	30	10.5	9.7E-23	8.6E-20
GOTERM_BP_ALL	nuclear-transcribed mRNA catabolic process. nonsense-mediated decay	26	9.1	3.4E-22	2.5E-19
GOTERM_BP_ALL	cytoplasmic translation	27	9.5	5.2E-21	3.3E-18
GOTERM_BP_ALL	protein localization to endoplasmic reticulum	27	9.5	8.6E-21	4.8E-18
GOTERM_BP_ALL	protein targeting to membrane	31	10.9	1.1E-20	5.4E-18
GOTERM_BP_ALL	nuclear-transcribed mRNA catabolic process	30	10.5	3.3E-20	1.5E-17
GOTERM_BP_ALL	viral gene expression	31	10.9	3.7E-20	1.5E-17
GOTERM_BP_ALL	translational initiation	28	9.8	1.2E-19	4.3E-17
GOTERM_BP_ALL	RNA catabolic process	32	11.2	4.8E-19	1.6E-16
GOTERM_BP_ALL	multi-organism metabolic process	31	10.9	7.5E-19	2.4E-16
GOTERM_BP_ALL	mRNA catabolic process	30	10.5	9.0E-19	2.7E-16
GOTERM_BP_ALL	establishment of protein localization to organelle	41	14.4	3.5E-17	9.8E-15
GOTERM_BP_ALL	protein targeting	42	14.7	4.8E-17	1.3E-14
GOTERM_BP_ALL	nucleobase-containing compound catabolic process	35	12.3	9.5E-17	2.4E-14
GOTERM_BP_ALL	establishment of protein localization to membrane	33	11.6	5.7E-16	1.3E-13
GOTERM_BP_ALL	viral life cycle	39	13.7	1.4E-15	3.2E-13
GOTERM_BP_ALL	heterocycle catabolic process	35	12.3	4.9E-15	1.0E-12
GOTERM_BP_ALL	cellular nitrogen compound catabolic process	35	12.3	5.9E-15	1.2E-12
GOTERM_BP_ALL	aromatic compound catabolic process	35	12.3	1.2E-14	2.3E-12
GOTERM_BP_ALL	protein localization to membrane	41	14.4	1.8E-14	3.3E-12
GOTERM_BP_ALL	intracellular transport	69	24.2	6.8E-14	1.2E-11
GOTERM_BP_ALL	viral process	55	19.3	7.4E-14	1.3E-11
GOTERM_BP_ALL	organic cyclic compound catabolic process	35	12.3	9.5E-14	1.6E-11
GOTERM_BP_ALL	symbiosis. encompassing mutualism through parasitism	56	19.6	1.1E-13	1.8E-11
GOTERM_BP_ALL	multi-organism cellular process	55	19.3	1.3E-13	2.1E-11
GOTERM_BP_ALL	interspecies interaction between organisms	56	19.6	1.5E-13	2.3E-11
GOTERM_BP_ALL	intracellular protein transport	52	18.2	2.8E-13	4.1E-11
GOTERM_BP_ALL	cellular protein localization	71	24.9	3.2E-13	4.5E-11
GOTERM_BP_ALL	cellular macromolecule localization	71	24.9	4.4E-13	5.9E-11
GOTERM_BP_ALL	cellular localization	90	31.6	4.9E-13	6.4E-11
GOTERM_BP_ALL	protein localization to organelle	47	16.5	8.1E-13	1.0E-10

GOTERM_BP_ALL	establishment of localization in cell	74	26.0	1.6E-12	2.0E-10
GOTERM_BP_ALL	establishment of protein localization	68	23.9	1.2E-11	1.4E-9
GOTERM_BP_ALL	establishment of localization	127	44.6	1.9E-11	2.2E-9
GOTERM_BP_ALL	peptide transport	65	22.8	5.5E-11	6.3E-9
GOTERM_BP_ALL	transport	123	43.2	7.1E-11	7.9E-9
GOTERM_BP_ALL	amide transport	65	22.8	1.2E-10	1.3E-8
GOTERM_BP_ALL	protein transport	63	22.1	1.4E-10	1.5E-8
GOTERM_BP_ALL	localization	148	51.9	2.2E-10	2.3E-8
GOTERM_BP_ALL	cellular macromolecule catabolic process	46	16.1	2.8E-10	2.9E-8
GOTERM_BP_ALL	protein localization	79	27.7	3.4E-10	3.3E-8
GOTERM_BP_ALL	cellular catabolic process	70	24.6	4.1E-10	4.0E-8
GOTERM_BP_ALL	multi-organism process	81	28.4	1.8E-9	1.7E-7
GOTERM_BP_ALL	ribosome biogenesis	23	8.1	2.5E-9	2.3E-7
GOTERM_BP_ALL	nitrogen compound transport	68	23.9	2.7E-9	2.4E-7
GOTERM_BP_ALL	organic substance transport	76	26.7	3.0E-9	2.7E-7
GOTERM_BP_ALL	rRNA processing	20	7.0	5.4E-9	4.7E-7
GOTERM_BP_ALL	rRNA metabolic process	21	7.4	8.5E-9	7.3E-7
GOTERM_BP_ALL	mRNA metabolic process	35	12.3	1.0E-8	8.8E-7
GOTERM_BP_ALL	macromolecule localization	82	28.8	1.5E-8	1.2E-6
GOTERM_BP_ALL	catabolic process	73	25.6	2.9E-8	2.3E-6
GOTERM_BP_ALL	macromolecule catabolic process	47	16.5	4.0E-8	3.1E-6
GOTERM_BP_ALL	cellular component organization or biogenesis	145	50.9	5.2E-8	4.1E-6
GOTERM_BP_ALL	cellular component biogenesis	85	29.8	1.5E-7	1.2E-5
GOTERM_BP_ALL	ribonucleoprotein complex biogenesis	26	9.1	2.2E-7	1.6E-5
GOTERM_BP_ALL	organic cyclic compound biosynthetic process	101	35.4	8.5E-7	6.3E-5
GOTERM_BP_ALL	nucleobase-containing compound biosynthetic process	97	34.0	9.1E-7	6.7E-5
GOTERM_BP_ALL	heterocycle biosynthetic process	98	34.4	1.1E-6	7.6E-5
GOTERM_BP_ALL	organelle organization	94	33.0	1.2E-6	8.5E-5
GOTERM_BP_ALL	negative regulation of macromolecule metabolic process	80	28.1	1.2E-6	8.5E-5
GOTERM_BP_ALL	aromatic compound biosynthetic process	97	34.0	2.2E-6	1.5E-4
GOTERM_BP_ALL	ncRNA processing	21	7.4	3.4E-6	2.3E-4
GOTERM_BP_ALL	organic substance catabolic process	58	20.4	3.6E-6	2.4E-4
GOTERM_BP_ALL	cellular protein metabolic process	119	41.8	4.0E-6	2.6E-4
GOTERM_BP_ALL	RNA biosynthetic process	88	30.9	4.0E-6	2.6E-4
GOTERM_BP_ALL	negative regulation of metabolic process	82	28.8	5.4E-6	3.4E-4
GOTERM_BP_ALL	nucleobase-containing compound metabolic process	127	44.6	5.8E-6	3.6E-4
GOTERM_BP_ALL	ncRNA metabolic process	25	8.8	6.7E-6	4.2E-4
GOTERM_BP_ALL	negative regulation of gene expression	63	22.1	8.6E-6	5.2E-4

GOTERM_BP_ALL	cell activation	44	15.4	9.2E-6	5.5E-4
GOTERM_BP_ALL	leukocyte activation involved in immune response	27	9.5	9.2E-6	5.5E-4
GOTERM_BP_ALL	cell activation involved in immune response	27	9.5	1.0E-5	5.9E-4
GOTERM_BP_ALL	regulation of metabolic process	142	49.8	1.0E-5	5.9E-4
GOTERM_BP_ALL	protein metabolic process	127	44.6	1.1E-5	6.3E-4
GOTERM_BP_ALL	organic cyclic compound metabolic process	133	46.7	1.1E-5	6.3E-4
GOTERM_BP_ALL	cellular macromolecular complex assembly	39	13.7	1.2E-5	6.5E-4
GOTERM_BP_ALL	nuclear import	14	4.9	1.3E-5	7.3E-4
GOTERM_BP_ALL	metabolic process	211	74.0	1.4E-5	7.4E-4
GOTERM_BP_ALL	nucleocytoplasmic transport	19	6.7	1.4E-5	7.6E-4
GOTERM_BP_ALL	nuclear transport	19	6.7	1.6E-5	8.5E-4
GOTERM_BP_ALL	myeloid leukocyte activation	25	8.8	1.6E-5	8.5E-4
GOTERM_BP_ALL	heterocycle metabolic process	128	44.9	1.6E-5	8.5E-4
GOTERM_BP_ALL	leukocyte activation	40	14.0	1.8E-5	9.2E-4
GOTERM_BP_ALL	immune effector process	40	14.0	2.1E-5	1.1E-3
GOTERM_BP_ALL	organonitrogen compound metabolic process	70	24.6	2.4E-5	1.2E-3
GOTERM_BP_ALL	positive regulation of signal transduction	46	16.1	3.4E-5	1.7E-3
GOTERM_BP_ALL	cellular aromatic compound metabolic process	127	44.6	4.0E-5	2.0E-3
GOTERM_BP_ALL	purine nucleoside triphosphate metabolic process	17	6.0	4.2E-5	2.0E-3
GOTERM_BP_ALL	amide biosynthetic process	40	14.0	4.6E-5	2.2E-3
GOTERM_BP_ALL	ATP metabolic process	16	5.6	4.6E-5	2.2E-3
GOTERM_BP_ALL	regulation of transport	48	16.8	4.8E-5	2.3E-3
GOTERM_BP_ALL	purine nucleoside monophosphate metabolic process	17	6.0	5.4E-5	2.5E-3
GOTERM_BP_ALL	purine ribonucleoside monophosphate metabolic process	17	6.0	5.4E-5	2.5E-3
GOTERM_BP_ALL	ribosomal large subunit biogenesis	8	2.8	6.1E-5	2.8E-3
GOTERM_BP_ALL	primary metabolic process	198	69.5	6.4E-5	2.9E-3
GOTERM_BP_ALL	regulation of nucleocytoplasmic transport	11	3.9	6.8E-5	3.0E-3
GOTERM_BP_ALL	cellular metabolic process	199	69.8	7.0E-5	3.1E-3
GOTERM_BP_ALL	cellular response to organic substance	64	22.5	7.3E-5	3.2E-3
GOTERM_BP_ALL	translation	37	13.0	7.4E-5	3.2E-3
GOTERM_BP_ALL	cellular response to stress	53	18.6	7.4E-5	3.2E-3
GOTERM_BP_ALL	regulation of intracellular transport	20	7.0	7.5E-5	3.2E-3
GOTERM_BP_ALL	nucleoside triphosphate metabolic process	17	6.0	7.9E-5	3.3E-3
GOTERM_BP_ALL	ribonucleoside monophosphate metabolic process	17	6.0	9.2E-5	3.8E-3
GOTERM_BP_ALL	macromolecular complex assembly	48	16.8	9.4E-5	3.9E-3
GOTERM_BP_ALL	organic substance biosynthetic process	127	44.6	9.8E-5	4.0E-3
GOTERM_BP_ALL	cellular component organization	129	45.3	9.9E-5	4.0E-3
GOTERM_BP_ALL	cristae formation	6	2.1	1.1E-4	4.2E-3

GOTERM_BP_ALL	peptide biosynthetic process	37	13.0	1.1E-4	4.5E-3
GOTERM_BP_ALL	regulation of signal transduction	72	25.3	1.2E-4	4.5E-3
GOTERM_BP_ALL	protein import into nucleus	12	4.2	1.2E-4	4.5E-3
GOTERM_BP_ALL	protein targeting to nucleus	12	4.2	1.2E-4	4.5E-3
GOTERM_BP_ALL	single-organism nuclear import	12	4.2	1.2E-4	4.5E-3
GOTERM_BP_ALL	purine ribonucleoside triphosphate metabolic process	16	5.6	1.3E-4	4.9E-3
GOTERM_BP_ALL	cellular protein complex assembly	24	8.4	1.3E-4	5.1E-3
GOTERM_BP_ALL	organic substance metabolic process	202	70.9	1.4E-4	5.1E-3
GOTERM_BP_ALL	nucleoside monophosphate metabolic process	17	6.0	1.5E-4	5.4E-3
GOTERM_BP_ALL	cellular biosynthetic process	125	43.9	1.5E-4	5.6E-3
GOTERM_BP_ALL	ribonucleoside triphosphate metabolic process	16	5.6	1.6E-4	5.8E-3
GOTERM_BP_ALL	organonitrogen compound biosynthetic process	50	17.5	1.6E-4	5.8E-3
GOTERM_BP_ALL	regulation of signal transduction by p53 class mediator	12	4.2	1.7E-4	6.0E-3
GOTERM_BP_ALL	mitochondrial ATP synthesis coupled proton transport	5	1.8	1.7E-4	6.0E-3
GOTERM_BP_ALL	regulation of intracellular protein transport	14	4.9	1.7E-4	6.1E-3
GOTERM_BP_ALL	positive regulation of cell communication	47	16.5	1.8E-4	6.2E-3
GOTERM_BP_ALL	cellular response to chemical stimulus	72	25.3	1.8E-4	6.2E-3
GOTERM_BP_ALL	leukocyte degranulation	20	7.0	1.8E-4	6.2E-3
GOTERM_BP_ALL	positive regulation of signaling	47	16.5	1.9E-4	6.6E-3
GOTERM_BP_ALL	regulation of peptide transport	22	7.7	1.9E-4	6.6E-3
GOTERM_BP_ALL	immune system process	74	26.0	2.0E-4	6.7E-3
GOTERM_BP_ALL	macromolecule biosynthetic process	109	38.2	2.0E-4	6.8E-3
GOTERM_BP_ALL	biosynthetic process	127	44.6	2.1E-4	6.9E-3
GOTERM_BP_ALL	positive regulation of intracellular transport	13	4.6	2.1E-4	6.9E-3
GOTERM_BP_ALL	regulation of autophagy	16	5.6	2.1E-4	6.9E-3
GOTERM_BP_ALL	positive regulation of response to stimulus	59	20.7	2.2E-4	7.2E-3
GOTERM_BP_ALL	DNA geometric change	9	3.2	2.3E-4	7.4E-3
GOTERM_BP_ALL	regulation of protein transport	21	7.4	2.4E-4	7.7E-3
GOTERM_BP_ALL	negative regulation of biological process	116	40.7	2.4E-4	7.8E-3
GOTERM_BP_ALL	granulocyte activation	19	6.7	2.6E-4	8.1E-3
GOTERM_BP_ALL	regulation of binding	16	5.6	2.7E-4	8.5E-3
GOTERM_BP_ALL	CD4-positive. alpha-beta T cell differentiation involved in immune response	7	2.5	2.8E-4	8.7E-3
GOTERM_BP_ALL	macromolecular complex subunit organization	53	18.6	2.8E-4	8.8E-3
GOTERM_BP_ALL	cellular macromolecule biosynthetic process	107	37.5	3.0E-4	9.1E-3
GOTERM_BP_ALL	alpha-beta T cell differentiation involved in immune response	7	2.5	3.0E-4	9.2E-3
GOTERM_BP_ALL	alpha-beta T cell activation involved in immune response	7	2.5	3.0E-4	9.2E-3
GOTERM_BP_ALL	myeloid leukocyte mediated immunity	20	7.0	3.2E-4	9.5E-3
GOTERM_BP_ALL	regulation of cellular catabolic process	24	8.4	3.2E-4	9.5E-3

GOTERM_BP_ALL	purine nucleotide metabolic process	20	7.0	3.3E-4	9.8E-3
GOTERM_BP_ALL	positive regulation of intracellular signal transduction	32	11.2	3.4E-4	9.8E-3
GOTERM_BP_ALL	cellular nitrogen compound biosynthetic process	105	36.8	3.4E-4	9.8E-3
GOTERM_BP_ALL	nucleic acid metabolic process	110	38.6	3.4E-4	9.8E-3
GOTERM_BP_ALL	cellular process	259	90.9	3.4E-4	1.0E-2
GOTERM_BP_ALL	regulation of intracellular signal transduction	48	16.8	3.5E-4	1.0E-2
GOTERM_BP_ALL	oxidative phosphorylation	9	3.2	3.6E-4	1.0E-2
GOTERM_BP_ALL	cellular amide metabolic process	43	15.1	3.7E-4	1.1E-2
GOTERM_BP_ALL	regulation of DNA binding	9	3.2	4.0E-4	1.1E-2
GOTERM_BP_ALL	regulation of macromolecule metabolic process	126	44.2	4.1E-4	1.1E-2
GOTERM_BP_ALL	peptidyl-threonine modification	9	3.2	4.6E-4	1.3E-2
GOTERM_BP_ALL	energy coupled proton transport. down electrochemical gradient	5	1.8	4.6E-4	1.3E-2
GOTERM_BP_ALL	ATP synthesis coupled proton transport	5	1.8	4.6E-4	1.3E-2
GOTERM_BP_ALL	phosphate-containing compound metabolic process	70	24.6	5.1E-4	1.4E-2
GOTERM_BP_ALL	regulation of response to biotic stimulus	11	3.9	5.1E-4	1.4E-2
GOTERM_BP_ALL	protein import	13	4.6	5.4E-4	1.5E-2
GOTERM_BP_ALL	positive regulation of transport	28	9.8	5.7E-4	1.5E-2
GOTERM_BP_ALL	CD4-positive, alpha-beta T cell activation	8	2.8	5.9E-4	1.6E-2
GOTERM_BP_ALL	leukocyte mediated immunity	27	9.5	6.2E-4	1.6E-2
GOTERM_BP_ALL	regulation of establishment of protein localization	21	7.4	6.2E-4	1.6E-2
GOTERM_BP_ALL	neutrophil activation	18	6.3	6.3E-4	1.7E-2
GOTERM_BP_ALL	peptide metabolic process	38	13.3	6.4E-4	1.7E-2
GOTERM_BP_ALL	T cell differentiation involved in immune response	7	2.5	6.4E-4	1.7E-2
GOTERM_BP_ALL	single-organism metabolic process	86	30.2	6.5E-4	1.7E-2
GOTERM_BP_ALL	purine ribonucleotide metabolic process	19	6.7	6.5E-4	1.7E-2
GOTERM_BP_ALL	single-organism cellular localization	29	10.2	6.6E-4	1.7E-2
GOTERM_BP_ALL	regulation of localization	62	21.8	6.8E-4	1.7E-2
GOTERM_BP_ALL	myeloid cell activation involved in immune response	19	6.7	6.8E-4	1.7E-2
GOTERM_BP_ALL	regulation of cell communication	75	26.3	6.8E-4	1.7E-2
GOTERM_BP_ALL	hydrogen ion transmembrane transport	9	3.2	7.5E-4	1.9E-2
GOTERM_BP_ALL	regulation of phosphate metabolic process	44	15.4	7.9E-4	1.9E-2
GOTERM_BP_ALL	purine-containing compound metabolic process	20	7.0	8.1E-4	2.0E-2
GOTERM_BP_ALL	cellular response to cytokine stimulus	31	10.9	8.4E-4	2.1E-2
GOTERM_BP_ALL	cellular nitrogen compound metabolic process	136	47.7	8.5E-4	2.1E-2
GOTERM_BP_ALL	regulation of phosphorus metabolic process	44	15.4	8.6E-4	2.1E-2
GOTERM_BP_ALL	regulation of response to stimulus	89	31.2	8.9E-4	2.1E-2
GOTERM_BP_ALL	ribonucleotide metabolic process	19	6.7	9.3E-4	2.2E-2
GOTERM_BP_ALL	regulation of signaling	75	26.3	9.5E-4	2.3E-2

GOTERM_BP_ALL	regulation of phosphorylation	40	14.0	9.5E-4	2.3E-2
GOTERM_BP_ALL	alpha-beta T cell differentiation	8	2.8	9.7E-4	2.3E-2
GOTERM_BP_ALL	cellular component assembly	66	23.2	9.7E-4	2.3E-2
GOTERM_BP_ALL	response to stress	86	30.2	1.0E-3	2.4E-2
GOTERM_BP_ALL	CD4-positive. alpha-beta T cell differentiation	7	2.5	1.0E-3	2.4E-2
GOTERM_BP_ALL	neutrophil degranulation	17	6.0	1.1E-3	2.4E-2
GOTERM_BP_ALL	protein complex assembly	33	11.6	1.1E-3	2.5E-2
GOTERM_BP_ALL	protein complex biogenesis	33	11.6	1.1E-3	2.5E-2
GOTERM_BP_ALL	phosphorus metabolic process	70	24.6	1.1E-3	2.5E-2
GOTERM_BP_ALL	regulation of protein modification process	44	15.4	1.2E-3	2.6E-2
GOTERM_BP_ALL	apoptotic signaling pathway	20	7.0	1.2E-3	2.6E-2
GOTERM_BP_ALL	T cell activation involved in immune response	8	2.8	1.2E-3	2.7E-2
GOTERM_BP_ALL	neutrophil activation involved in immune response	17	6.0	1.2E-3	2.7E-2
GOTERM_BP_ALL	regulation of protein targeting	10	3.5	1.2E-3	2.7E-2
GOTERM_BP_ALL	positive regulation of apoptotic signaling pathway	10	3.5	1.2E-3	2.7E-2
GOTERM_BP_ALL	inner mitochondrial membrane organization	6	2.1	1.2E-3	2.7E-2
GOTERM_BP_ALL	regulation of adaptive immune response	10	3.5	1.3E-3	2.8E-2
GOTERM_BP_ALL	positive regulation of cytokine production	18	6.3	1.3E-3	2.8E-2
GOTERM_BP_ALL	response to biotic stimulus	32	11.2	1.3E-3	2.8E-2
GOTERM_BP_ALL	immune response	54	18.9	1.3E-3	2.8E-2
GOTERM_BP_ALL	response to other organism	31	10.9	1.3E-3	2.8E-2
GOTERM_BP_ALL	ribose phosphate metabolic process	19	6.7	1.3E-3	2.9E-2
GOTERM_BP_ALL	response to external biotic stimulus	31	10.9	1.4E-3	2.9E-2
GOTERM_BP_ALL	regulation of symbiosis. encompassing mutualism through parasitism	14	4.9	1.4E-3	2.9E-2
GOTERM_BP_ALL	single-organism organelle organization	44	15.4	1.4E-3	3.0E-2
GOTERM_BP_ALL	phosphorylation	53	18.6	1.5E-3	3.0E-2
GOTERM_BP_ALL	cellular response to oxygen-containing compound	31	10.9	1.5E-3	3.1E-2
GOTERM_BP_ALL	positive regulation of protein phosphorylation	28	9.8	1.5E-3	3.1E-2
GOTERM_BP_ALL	positive regulation of molecular function	41	14.4	1.5E-3	3.1E-2
GOTERM_BP_ALL	neutrophil mediated immunity	17	6.0	1.6E-3	3.2E-2
GOTERM_BP_ALL	regulation of macroautophagy	10	3.5	1.6E-3	3.2E-2
GOTERM_BP_ALL	regulation of organelle organization	35	12.3	1.6E-3	3.3E-2
GOTERM_BP_ALL	autophagy	18	6.3	1.7E-3	3.3E-2
GOTERM_BP_ALL	nitrogen compound metabolic process	141	49.5	1.7E-3	3.4E-2
GOTERM_BP_ALL	organophosphate metabolic process	31	10.9	1.7E-3	3.4E-2
GOTERM_BP_ALL	exocytosis	25	8.8	1.7E-3	3.4E-2
GOTERM_BP_ALL	defense response to other organism	21	7.4	1.7E-3	3.4E-2
GOTERM_BP_ALL	ion transport	40	14.0	1.7E-3	3.4E-2

GOTERM_BP_ALL	regulation of molecular function	65	22.8	1.8E-3	3.4E-2
GOTERM_BP_ALL	proton transport	9	3.2	1.8E-3	3.4E-2
GOTERM_BP_ALL	hydrogen transport	9	3.2	1.8E-3	3.4E-2
GOTERM_BP_ALL	neutrophil homeostasis	4	1.4	1.8E-3	3.4E-2
GOTERM_BP_ALL	positive regulation of phosphate metabolic process	31	10.9	1.8E-3	3.4E-2
GOTERM_BP_ALL	positive regulation of phosphorus metabolic process	31	10.9	1.8E-3	3.4E-2
GOTERM_BP_ALL	T-helper cell differentiation	6	2.1	1.8E-3	3.4E-2
GOTERM_BP_ALL	cellular protein modification process	82	28.8	1.8E-3	3.4E-2
GOTERM_BP_ALL	protein modification process	82	28.8	1.8E-3	3.4E-2
GOTERM_BP_ALL	RNA metabolic process	98	34.4	1.8E-3	3.4E-2
GOTERM_BP_ALL	immune system development	28	9.8	1.8E-3	3.4E-2
GOTERM_BP_ALL	alpha-beta T cell activation	9	3.2	1.8E-3	3.4E-2
GOTERM_BP_ALL	response to bacterium	23	8.1	1.9E-3	3.5E-2
GOTERM_BP_ALL	lymphocyte activation involved in immune response	10	3.5	1.9E-3	3.5E-2
GOTERM_BP_ALL	cell chemotaxis	13	4.6	1.9E-3	3.5E-2
GOTERM_BP_ALL	positive regulation of phosphorylation	29	10.2	1.9E-3	3.5E-2
GOTERM_BP_ALL	nucleobase-containing small molecule metabolic process	22	7.7	1.9E-3	3.6E-2
GOTERM_BP_ALL	positive regulation of MAPK cascade	18	6.3	2.0E-3	3.7E-2
GOTERM_BP_ALL	regulation of transferase activity	27	9.5	2.0E-3	3.7E-2
GOTERM_BP_ALL	positive regulation of immune system process	32	11.2	2.1E-3	3.8E-2
GOTERM_BP_ALL	regulation of interleukin-1 production	8	2.8	2.2E-3	3.9E-2
GOTERM_BP_ALL	positive regulation of intrinsic apoptotic signaling pathway	6	2.1	2.2E-3	4.0E-2
GOTERM_BP_ALL	regulation of apoptotic signaling pathway	15	5.3	2.3E-3	4.1E-2
GOTERM_BP_ALL	protein localization to nucleus	13	4.6	2.3E-3	4.2E-2
GOTERM_BP_ALL	single-organism intracellular transport	22	7.7	2.5E-3	4.5E-2
GOTERM_BP_ALL	T cell activation	17	6.0	2.5E-3	4.5E-2
GOTERM_BP_ALL	regulation of catalytic activity	53	18.6	2.6E-3	4.6E-2
GOTERM_BP_ALL	protein complex subunit organization	37	13.0	2.6E-3	4.6E-2
GOTERM_BP_ALL	cell surface receptor signaling pathway	64	22.5	2.7E-3	4.7E-2
GOTERM_BP_ALL	response to oxygen-containing compound	40	14.0	2.7E-3	4.7E-2
GOTERM_BP_ALL	regulation of viral process	13	4.6	2.8E-3	4.8E-2
GOTERM_BP_ALL	cellular macromolecule metabolic process	163	57.2	2.8E-3	4.8E-2

Supplementary Table 5. Results of all the analyses of those 16 genes selected for the validation phase.

		DATA FROM DISCOVERY PHASE (with RNA-Seq) $												DATA FROM VALIDATION PHASE (with OpenArray)&							
Target		dnPD vs HCg			dnPD vs Cent			Cent vs Hci			HCg vs Hci			adPD vs HCs			PD-sib vs HCs			Target	
ensembl_gene_ID/ transcript ID	Name	log2 FoldChange	P-value adj	Result	log2 FoldChange	P-value adj	Result	log2 FoldChange	P-value adj	Result	log2 FoldChange	P-value adj	Result	log2 FoldChange	P-value adj	Result	log2 FoldChange	P-value adj	Result	Name	Assay ID
ENSG00000119408	NEK6	0.3960	5.54E-04	up-regulated	-0.2172	3.55E-01	non- significative diferences	-0.0090	9.94E-01	non- significative diferences	-0.6233	1.20E-03	down-regulated	0.1309	6.39E-01	Insignificant	0.7681	3.80E-23	Flat	NEK6	Hs01032395
ENSG00000117139	KDM5B	0.2440	7.96E-03	up-regulated	-0.0218	9.12E-01	non- significative diferences	-0.3002	2.58E-01	non- significative diferences	-0.5330	2.95E-05	down-regulated	0.1865	2.75E-01	Insignificant	0.9123	3.70E-28	Flat	KDM5B	Hs00981910
ENSG00000170322	NFRKB	0.2601	1.79E-02	up-regulated	0.0563	8.08E-01	non- significative diferences	-0.2458	1.59E-01	non- significative diferences	-0.4342	3.14E-03	down-regulated	0.0000	9.84E-01	Insignificant	1.0122	4.30E-23	up-regulated	NFRKB	Hs01123207
ENSG00000127507	ADGRE2	0.3899	1.79E-02	up-regulated	0.1185	8.05E-01	non- significative diferences	-0.4551	1.61E-01	non- significative diferences	-0.7283	7.04E-04	down-regulated	0.2079	2.14E-01	Insignificant	1.2594	3.30E-42	up-regulated	ADGRE2	Hs01126919
ENSG00000061273	HDAC7	0.3287	3.31E-02	up-regulated	-0.1014	7.12E-01	non- significative diferences	-0.4568	1.49E-01	non- significative diferences	-0.8621	5.18E-05	down-regulated	-0.0130	1.00E+00	Insignificant	0.6098	6.70E-16	Flat	HDAC7	Hs01045864
ENSG00000110395	CBL	0.3641	3.31E-02	up-regulated	-0.2468	3.85E-01	non- significative diferences	-0.4808	1.91E-01	non- significative diferences	-1.1207	2.75E-05	down-regulated	0.1814	7.05E-01	Insignificant	0.7102	4.80E-08	Flat	CBL	Hs01011446
ENSG00000163932	PRKCD	0.3597	3.40E-02	up-regulated	-0.0198	8.68E-01	non- significative diferences	-0.3865	3.81E-01	non- significative diferences	-0.8011	2.23E-03	down-regulated	0.0909	7.05E-01	Insignificant	0.9971	1.40E-38	Flat	PRKCD	Hs01090047
ENSG00000072786	STK10	0.2698	3.66E-02	up-regulated	-0.0453	8.48E-01	non- significative diferences	-0.4317	1.00E-01	non- significative diferences	-0.7157	1.47E-04	down-regulated	0.0370	1.00E+00	Insignificant	1.7727	0.00E+00	up-regulated	STK10	Hs00950481
ENSG00000180694	TMEM64	-0.2282	3.78E-02	down-regulated	-0.1230	5.45E-01	non- significative diferences	0.1857	3.70E-01	non- significative diferences	0.2950	9.24E-02	non- significative diferences	0.0000	1.05E+00	Insignificant	0.0272	1.00E+00	Insignificant	TMEM64	Hs01595139
ENSG00000164300	SERINC5	0.2413	4.12E-02	up-regulated	0.3171	1.61E-01	non- significative diferences	-0.6059	3.31E-02	down-regulated	-0.6085	3.94E-04	down-regulated	-0.0970	9.19E-01	Insignificant	0.6544	1.70E-14	Flat	SERINC5	Hs00968169
ENSG00000140443	IGF1R	0.2878	4.24E-02	up-regulated	0.0916	8.20E-01	non- significative diferences	-0.5418	1.31E-01	non- significative diferences	-0.8127	1.44E-04	down-regulated	0.1362	6.39E-01	Insignificant	1.1808	1.10E-37	up-regulated	IGF1R	Hs00609566
ENSG00000182749	PAQR7	0.2324	4.25E-02	up-regulated	0.4267	5.18E-02	non- significative diferences	-0.2745	2.99E-01	non- significative diferences	-0.1428	3.31E-01	non- significative diferences	0.1712	6.39E-01	Insignificant	1.0482	8.10E-23	up-regulated	PAQR7	Hs00753107
ENSG00000171033	PKIA	-0.2914	4.27E-02	down-regulated	0.5201	3.08E-02	up-regulated	-0.1893	6.17E-01	non- significative diferences	0.6190	6.84E-03	up-regulated	-0.0233	9.55E-01	Insignificant	-0.0634	1.00E+00	Insignificant	PKIA	Hs00738983
**ENSG00000275395**	**FCGBP**	**0.3563**	**4.62E-02**	**up-regulated**	**0.6967**	**8.82E-03**	**up-regulated**	**-0.4490**	**1.83E-01**	**non- significative diferences**	**-0.2837**	**3.46E-01**	**non- significative diferences**	**1.1230**	**4.10E-02**	**up-regulated**	**1.1953**	**8.60E-05**	**up-regulated**	**FCGBP**	**Hs00175398**
ENSG00000182774	RPS17	-0.3832	4.65E-02	down-regulated	-0.0563	9.20E-01	non- significative diferences	0.7480	3.19E-02	up-regulated	1.1355	1.11E-03	up-regulated	-0.0893	1.00E+00	Insignificant	-4.1078	0.00E+00	down-regulated	RPS17	Hs00734303
ENSG00000148400	NOTCH1	0.3150	5.61E-02	non- significative diferences	-0.0691	8.63E-01	non- significative diferences	-0.2952	4.50E-01	non- significative diferences	-0.7296	9.53E-03	down-regulated	0.1296	6.85E-01	Insignificant	1.5558	0.00E+00	up-regulated	NOTCH1	Hs01062014

$: Analysis with the statistical software R and the Bioconductor package DESeq2. Differences were considered statistically significant when the P-value adjusted by multiple testing correction (using the Benjamin-Hochberg method) was <0.05.

&: Analysis with the Thermo Fisher Cloud software, based on FDR adjusted p-value. The result “Insignificant” or “Flat” is reported by the software when the log2FC is <1 and >−1 and/or when the FDR adjusted p-value is >0.05.

Supplementary Table 3. PROPAG-AGEING consortium members.

Name (Surname, first name)	Partner	Affiliation
Adarmes-Gómez, Astrid	SAS	Unidad de Trastornos del Movimiento, Servicio de Neurología y Neurofisiología Clínica, Instituto de Biomedicina de Sevilla, Hospital Universitario Virgen del Rocío/CSIC/Universidad de Sevilla, Seville, Spain
Azevedo, Tiago	UCAM	Department of Computer Science and Technology, University of Cambridge, Cambridge, United Kingdom.
Bacalini, Maria Giulia	AUSL	IRCCS Istituto delle Scienze Neurologiche di Bologna, Bologna, Italy
Baldelli, Luca	AUSL	Department of Biomedical and NeuroMotor Sciences (DiBiNeM), University of Bologna, Italy
Bartoletti-Stella, Anna	AUSL	IRCCS Istituto delle Scienze Neurologiche di Bologna, Bologna, Italy
Bhatia, Kailash P.	UCL	Department of Clinical and Movement Neurosciences UCL Queen Square Institute of Neurology London United Kingdom
Bonilla-Toribio, Marta	SAS	Unidad de Trastornos del Movimiento, Servicio de Neurología y Neurofisiología Clínica, Instituto de Biomedicina de Sevilla, Hospital Universitario Virgen del Rocío/CSIC/Universidad de Sevilla, Seville, Spain
Boninsegna, Claudia	AUSL	IRCCS Istituto delle Scienze Neurologiche di Bologna, Bologna, Italy
Broli, Marcella	AUSL	IRCCS Istituto delle Scienze Neurologiche di Bologna, Bologna, Italy
Buiza-Rueda, Dolores	SAS	Unidad de Trastornos del Movimiento, Servicio de Neurología y Neurofisiología Clínica, Instituto de Biomedicina de Sevilla, Hospital Universitario Virgen del Rocío/CSIC/Universidad de Sevilla, Seville, Spain

Calandra-Buonaura, Giovanna	AUSL	Department of Biomedical and NeuroMotor Sciences (DiBiNeM), University of Bologna, Italy; IRCCS Istituto delle Scienze Neurologiche di Bologna, Bologna, Italy
Capellari, Sabina	AUSL	Department of Biomedical and NeuroMotor Sciences (DiBiNeM), University of Bologna, Italy; IRCCS Istituto delle Scienze Neurologiche di Bologna, Bologna, Italy
Cilea, Rosalia	AUSL	IRCCS Istituto delle Scienze Neurologiche di Bologna, Bologna, Italy
Clayton Robert	UCL	Centre for Inborn Errors of Metabolism, UCL Institute of Child Health, London, United Kingdom
Cortelli, Pietro	AUSL	Department of Biomedical and NeuroMotor Sciences (DiBiNeM), University of Bologna, Italy; IRCCS Istituto delle Scienze Neurologiche di Bologna, Bologna, Italy
Dal Molin, Alessandra	PG	Personal Genomics s.r.l
De Luca, Silvia	AUSL	IRCCS Istituto delle Scienze Neurologiche di Bologna, Bologna, Italy
De Massis, Patrizia	AUSL	Neurology Unit, Medical Oncological Department, S. Maria Della Scaletta Hospital, 40026, Imola, BO, Italy
Dimitri, Giovanna Maria	UCAM	Department of Computer Science and Technology, University of Cambridge, Cambridge, United Kingdom.
Doykov, Ivan	UCL	Centre for Inborn Errors of Metabolism, UCL Institute of Child Health Library, London, UK.
Fabbri, Giovanni	AUSL	Azienda Unità Sanitaria Locale di Bologna, ASL Bologna, Italy

Franceschi, Claudio	AUSL	IRCCS Istituto delle Scienze Neurologiche di Bologna, Bologna, Italy; Institute of Information Technologies, Mathematics and Mechanics, Lobachevsky University, Nizhniy Novgorod, Russia
Gabellini, Anna	AUSL	IRCCS Istituto delle Scienze Neurologiche di Bologna, Bologna, Italy
Garagnani, Paolo	UNIBO	Department of Experimental, Diagnostic, and Specialty Medicine (DIMES), University of Bologna, Bologna, Italy
Giuliani, Cristina	UNIBO	Department of Biological, Geological, and Environmental Sciences (BiGeA), Laboratory of Molecular Anthropology and Centre for Genome Biology, University of Bologna, Bologna, Italy; School of Anthropology and Museum Ethnography, University of Oxford, Oxford, United Kingdom; Alma Mater Research Institute on Global Challenges and Climate Change (Alma Climate), University of Bologna, Bologna, Italy
Gómez-Garre, Pilar	SAS	Unidad de Trastornos del Movimiento, Servicio de Neurología y Neurofisiología Clínica, Instituto de Biomedicina de Sevilla, Hospital Universitario Virgen del Rocío/CSIC/Universidad de Sevilla, Seville, Spain
Guaraldi, Pietro	AUSL	IRCCS Istituto delle Scienze Neurologiche di Bologna, Bologna, Italy
Hägg, Sara	KI	Department of Medical Epidemiology and Biostatistics, Karolinska Institutet, Stockholm, Sweden
Hällqvist, Jenny	UCL	Centre for Inborn Errors of Metabolism, UCL Institute of Child Health Library, London, UK.
Halsband, Claire	UMG	Department of Clinical Neurophysiology, University Medical Center Göttingen, Göttingen, Germany; Department of Gerontopsychiatry, Rhein-Mosel-Fachklinik, Andernach, Germany
Heywood, Wendy	UCL	Centre for Inborn Errors of Metabolism, UCL Institute of Child Health Library, London, UK; NIHR Great Ormond Street Biomedical Research Centre, Great Ormond Street Hospital and UCL Great Ormond Street Institute of Child Health, London, UK

Houlden, Henry	UCL	Department of Neuromuscular Disorders, UCL Queen Square Institute of Neurology, London, WC1N 3BG, UK
Jesús, Silvia	SAS	Unidad de Trastornos del Movimiento, Servicio de Neurología y Neurofisiología Clínica, Instituto de Biomedicina de Sevilla, Hospital Universitario Virgen del Rocío/CSIC/Universidad de Sevilla, Seville, Spain
Jylhävä, Juulia	KI	Department of Medical Epidemiology and Biostatistics, Karolinska Institutet, Stockholm, Sweden
Labrador-Espinosa, Miguel A.	SAS	Unidad de Trastornos del Movimiento, Servicio de Neurología y Neurofisiología Clínica, Instituto de Biomedicina de Sevilla, Hospital Universitario Virgen del Rocío/CSIC/Universidad de Sevilla, Seville, Spain
Licari, Cristina	CRMMP	CERM, University of Florence, Sesto Fiorentino, Florence, Italy
Liò, Pietro	UCAM	Department of Computer Science and Technology, University of Cambridge, Cambridge, United Kingdom.
Luchinat, Claudio	CRMMP	Centro Risonanze Magnetiche (CERM) and Department of Chemistry, University of Florence, Florence, Italy
Macias, Daniel	SAS	Unidad de Trastornos del Movimiento, Servicio de Neurología y Neurofisiología Clínica, Instituto de Biomedicina de Sevilla, Hospital Universitario Virgen del Rocío/CSIC/Universidad de Sevilla, Seville, Spain
Macrì, Stefania	AUSL	Casa di cura Villa Baruzziana, Bologna
Magrinelli, Francesca	UCL	Department of Clinical and Movement Neurosciences, Queen Square Institute of Neurology, University College London, London, UK; Department of Neurosciences, Biomedicine and Movement Sciences, University of Verona, Verona, Italy
Martín Rodríguez, Juan Francisco	SAS	Unidad de Trastornos del Movimiento, Servicio de Neurología y Neurofisiología Clínica, Instituto de Biomedicina de Sevilla, Hospital Universitario Virgen del Rocío/CSIC/Universidad de Sevilla, Seville, Spain

Massimo, Delledonne	PG	Department of Biotechnology, University of Verona, Strada Le Grazie 15, 37134, Verona, Italy
Maturo, Maria Giovanna	PG	Department of Biotechnological and Applied Clinical Sciences, University of L'Aquila, L'Aquila, Italy
Mengozzi, Giacomo	AUSL	IRCCS Istituto delle Scienze Neurologiche di Bologna, Bologna, Italy
Meoni, Gaia	CRMMP	Giotto Biotech srl, Florence, Italy
Mignani, Francesco	AUSL	IRCCS Istituto delle Scienze Neurologiche di Bologna, Bologna, Italy
Milazzo, Maddalena	UNIBO	Department of Experimental, Diagnostic, and Specialty Medicine (DIMES), University of Bologna, Bologna, Italy
Mills, Kevin	UCL	NIHR Great Ormond Street Biomedical Research Centre, Great Ormond Street Hospital and UCL Great Ormond Street Institute of Child Health, London, UK; Centre for Inborn Errors of Metabolism, UCL Institute of Child Health Library, London, UK.
Mir, Pablo	SAS	Unidad de Trastornos del Movimiento, Servicio de Neurología y Neurofisiología Clínica, Instituto de Biomedicina de Sevilla, Hospital Universitario Virgen del Rocío/CSIC/Universidad de Sevilla, Seville, Spain
Mollenhauer, Brit	UMG	Paracelsus-Elena-Klinik, Kassel, Germany; Department of Neurology, University Medical Centre Goettingen, Goettingen, Germany
Muñoz-Delgado, Laura	SAS	Unidad de Trastornos del Movimiento, Servicio de Neurología y Neurofisiología Clínica, Instituto de Biomedicina de Sevilla, Hospital Universitario Virgen del Rocío/CSIC/Universidad de Sevilla, Seville, Spain
Nardini, Christine	PG	Instituto per le Applicazioni del Calcolo Mauro Picone, CNR, Via dei Taurini, 19, Roma, Italy

Nassetti, Stefania Alessandra	AUSL	IRCCS Istituto delle Scienze Neurologiche di Bologna, Bologna, Italy
Pedersen, Nancy L.	KI	Department of Medical Epidemiology and Biostatistics, Karolinska Institutet, Stockholm, Sweden
Periñán-Tocino, Teresa	SAS	Unidad de Trastornos del Movimiento, Servicio de Neurología y Neurofisiología Clínica, Instituto de Biomedicina de Sevilla, Hospital Universitario Virgen del Rocío/CSIC/Universidad de Sevilla, Seville, Spain
Pineda-Sánchez, Rocio	SAS	Unidad de Trastornos del Movimiento, Servicio de Neurología y Neurofisiología Clínica, Instituto de Biomedicina de Sevilla, Hospital Universitario Virgen del Rocío/CSIC/Universidad de Sevilla, Seville, Spain
Pirazzini, Chiara	AUSL	IRCCS Istituto delle Scienze Neurologiche di Bologna, Bologna, Italy
Provini, Federica	AUSL	Department of Biomedical and NeuroMotor Sciences (DiBiNeM), University of Bologna, Italy; IRCCS Istituto delle Scienze Neurologiche di Bologna, Bologna, Italy
Ravaioli, Francesco	UNIBO	Department of Experimental, Diagnostic, and Specialty Medicine (DIMES), University of Bologna, Bologna, Italy
Sala, Claudia	UNIBO	Department of Physics and Astronomy, University of Bologna, Viale Berti Pichat 6/2, Bologna, Italy
Sambati, Luisa	AUSL	IRCCS Istituto delle Scienze Neurologiche di Bologna, Bologna, Italy
Scaglione, Cesa Lorella Maria	AUSL	IRCCS Istituto delle Scienze Neurologiche di Bologna, Bologna, Italy
Schade, Sebastian	UMG	Department of Clinical Neurophysiology University Medical Center Göttingen Göttingen Germany

Schreglmann, Sebastian	UCL	Department of Clinical and Movement Neurosciences UCL Queen Square Institute of Neurology London United Kingdom
Spasov, Simeon	UCAM	Department of Computer Science and Technology, University of Cambridge, Cambridge, United Kingdom.
Strom, Stephen	KI	Department of Laboratory Medicine, Karolinska Institute and Karolinska Universitetssjukhuset, 171 76, Stockholm, Sweden.
Tenori, Leonardo	CRMMP	Consorzio Interuniversitario Risonanze Magnetiche di Metalloproteine (CIRMMP), Florence, Italy
Trenkwalder, Claudia	UMG	Paracelsus-Elena-Klinik, Kassel, Germany; Department of Neurosurgery University Medical Center Göttingen Göttingen Germany
Turano, Paola	CRMMP	Centro Risonanze Magnetiche (CERM) and Department of Chemistry, University of Florence, Florence, Italy
Valzania, Franco	AUSL	Neurology Unit, Neuromotor & Rehabilitation Department, Azienda USL-IRCCS di Reggio Emilia, Viale Risorgimento 80, 42123, Reggio Emilia, Italy
Williams, Dylan	KI	Department of Medical Epidemiology and Biostatistics, Karolinska Institutet, Stockholm, Sweden
Xumerle, Luciano	PG	Personal Genomics s.r.l
Zago, Elisa	PG	Personal Genomics s.r.l
