# Supplementary material for: Transcriptomic analysis reveals an association of FCGBP with Parkinson’s disease
Source: NPJ Parkinsons Dis. 2022 Nov 12;8:157. doi: 10.1038/s41531-022-00415-7 (PMC9653420; doi:10.1038/s41531-022-00415-7)
Supplement: Supplementary file 2 — Reporting Summary [file 41531_2022_415_MOESM2_ESM.pdf]

nature portfolio

Corresponding author(s): Pablo Mir Rivera and Pilar Gómez-Garre

Last updated by author(s): 10-13-2022

Reporting Summary

Nature Portfolio wishes to improve the reproducibility of the work that we publish. This form provides structure for consistency and transparency in reporting. For further information on Nature Portfolio policies, see our Editorial Policies and the Editorial Policy Checklist.

Statistics

For all statistical analyses, confirm that the following items are present in the figure legend, table legend, main text, or Methods section.

n/a	Confirmed	
☐	☒	The exact sample size (*n*) for each experimental group/condition, given as a discrete number and unit of measurement
☐	☒	A statement on whether measurements were taken from distinct samples or whether the same sample was measured repeatedly
☐	☒	The statistical test(s) used AND whether they are one- or two-sided<br>*Only common tests should be described solely by name; describe more complex techniques in the Methods section.*
☐	☒	A description of all covariates tested
☐	☒	A description of any assumptions or corrections, such as tests of normality and adjustment for multiple comparisons
☐	☒	A full description of the statistical parameters including central tendency (e.g. means) or other basic estimates (e.g. regression coefficient) AND variation (e.g. standard deviation) or associated estimates of uncertainty (e.g. confidence intervals)
☐	☒	For null hypothesis testing, the test statistic (e.g. *F*, *t*, *r*) with confidence intervals, effect sizes, degrees of freedom and *P* value noted<br>*Give P values as exact values whenever suitable.*
☒	☐	For Bayesian analysis, information on the choice of priors and Markov chain Monte Carlo settings
☒	☐	For hierarchical and complex designs, identification of the appropriate level for tests and full reporting of outcomes
☒	☐	Estimates of effect sizes (e.g. Cohen's *d*, Pearson's *r*), indicating how they were calculated

Our web collection on statistics for biologists contains articles on many of the points above.

Software and code

Policy information about availability of computer code

Data collection	No software was used.
Data analysis	We aligned the sequence files against the hg38 reference genome with TopHat v.2.0.6 and obtained the number of reads at the gene level with HTSeq software v.0.6.0. Differential gene expression analyis was done in R v.3.6.0 using DESeq2.

For manuscripts utilizing custom algorithms or software that are central to the research but not yet described in published literature, software must be made available to editors and reviewers. We strongly encourage code deposition in a community repository (e.g. GitHub). See the Nature Portfolio guidelines for submitting code & software for further information.

Data

Policy information about availability of data

All manuscripts must include a data availability statement. This statement should provide the following information, where applicable:

- Accession codes, unique identifiers, or web links for publicly available datasets
- A description of any restrictions on data availability
- For clinical datasets or third party data, please ensure that the statement adheres to our policy

The data that support the findings of this study are available from the corresponding author upon reasonable request.

Human research participants

Policy information about studies involving human research participants and Sex and Gender in Research.

Reporting on sex and gender	Sex was included as covariate in the design formula, whereas other experimental covariates (such as age) were not considered since no differences were detected between groups.
Population characteristics	We analyzed 47 patients with de novo PD (those with no history of present or past therapy with anti-parkinsonian drugs), 58 healthy controls from Germany and 7 from Italy, as well as 10 healthy centenarians. For the second stage of validation, we established 3 independent cohorts: 201 patients with advanced PD (disease duration of at least 5 years) (2.5% patients carried pathogenic LRRK2 and PRKN mutations), 177 healthy controls and 340 PD-siblings from Spain, Germany, and Italy.
Recruitment	This study included participants from the multicenter PROPAG-AGEING project. Parkinson's disease (PD) was diagnosed by movement disorder neurologists according to the United Kingdom Parkinson's Disease Society Brain Bank criteria. The healthy controls, healthy centenarians, and siblings of patients with a diagnosis of sporadic PD had no active known/treated central nervous system condition, as determined during a clinical interview. The PD-siblings cohort was deeply characterized for several clinical parameters, with particular regard for motor and non-motor symptoms and video-polysomnography-confirmed REM sleep behavior disorder. Accurate evaluation of these parameters allowed estimating the risk of developing PD.
Ethics oversight	The study was approved by the local ethics committees of all centers participating in the consortium (UMG-GOE ethics committee approval no. 19/5/16 of August 2016, ISNB ethics committee approval no. 16018 of May 2016, SAS ethical committee approval no. 2014/PI173 of September 2016) and was conducted according to the principles expressed in the Declaration of Helsinki.

Note that full information on the approval of the study protocol must also be provided in the manuscript.

Field-specific reporting

Please select the one below that is the best fit for your research. If you are not sure, read the appropriate sections before making your selection.

☒ Life sciences ☐ Behavioural & social sciences ☐ Ecological, evolutionary & environmental sciences

For a reference copy of the document with all sections, see nature.com/documents/nr-reporting-summary-flat.pdf

Life sciences study design

All studies must disclose on these points even when the disclosure is negative.

Sample size	We analyzed 47 patients with de novo PD (those with no history of present or past therapy with anti-parkinsonian drugs), 58 healthy controls from Germany and 7 from Italy, as well as 10 healthy centenarians. For the second stage of validation, we established 3 independent cohorts: 201 patients with advanced PD (disease duration of at least 5 years) (2.5% patients carried pathogenic LRRK2 and PRKN mutations), 177 healthy controls and 340 PD-siblings from Spain, Germany, and Italy.
Data exclusions	No data were excluded from the analyses
Replication	We performed the validation of the DGE findings from the RNA-Seq analysis on a QuantStudioTM 12K Flex OpenArray® Real-Time PCR System (Thermo Fisher Scientific, CA, USA).
Randomization	We performed RNA sequencing from patients with de novo PD, centenarians, and healthy controls. The expression of the selected genes was validated using samples from independent cohorts of patients with advanced PD, healthy siblings of PD patients, as well as healthy controls.
Blinding	Each sample was assigned an alphanumeric ID to carry out an effective blinding, in order to maintain the information of the subjects of study in absolute confidentiality.

Reporting for specific materials, systems and methods

We require information from authors about some types of materials, experimental systems and methods used in many studies. Here, indicate whether each material, system or method listed is relevant to your study. If you are not sure if a list item applies to your research, read the appropriate section before selecting a response.

Materials & experimental systems

n/a	Involved in the study
☒	☐ Antibodies
☒	☐ Eukaryotic cell lines
☒	☐ Palaeontology and archaeology
☒	☐ Animals and other organisms
☒	☐ Clinical data
☒	☐ Dual use research of concern

Methods

n/a	Involved in the study
☒	☐ ChIP-seq
☒	☐ Flow cytometry
☒	☐ MRI-based neuroimaging
